# Oncolytic Viruses for Malignant Glioma: On the Verge of Success?

**DOI:** 10.3390/v13071294

**Published:** 2021-07-02

**Authors:** Yogesh R. Suryawanshi, Autumn J. Schulze

**Affiliations:** Department of Molecular Medicine, Mayo Clinic College of Medicine, Rochester, MN 55905, USA; suryawanshi.yogesh@mayo.edu

**Keywords:** glioblastoma, oncolytic virus, blood–brain barrier, tumor microenvironment, tumor heterogeneity

## Abstract

Glioblastoma is one of the most difficult tumor types to treat with conventional therapy options like tumor debulking and chemo- and radiotherapy. Immunotherapeutic agents like oncolytic viruses, immune checkpoint inhibitors, and chimeric antigen receptor T cells have revolutionized cancer therapy, but their success in glioblastoma remains limited and further optimization of immunotherapies is needed. Several oncolytic viruses have demonstrated the ability to infect tumors and trigger anti-tumor immune responses in malignant glioma patients. Leading the pack, oncolytic herpesvirus, first in its class, awaits an approval for treating malignant glioma from MHLW, the federal authority of Japan. Nevertheless, some major hurdles like the blood–brain barrier, the immunosuppressive tumor microenvironment, and tumor heterogeneity can engender suboptimal efficacy in malignant glioma. In this review, we discuss the current status of malignant glioma therapies with a focus on oncolytic viruses in clinical trials. Furthermore, we discuss the obstacles faced by oncolytic viruses in malignant glioma patients and strategies that are being used to overcome these limitations to (1) optimize delivery of oncolytic viruses beyond the blood–brain barrier; (2) trigger inflammatory immune responses in and around tumors; and (3) use multimodal therapies in combination to tackle tumor heterogeneity, with an end goal of optimizing the therapeutic outcome of oncolytic virotherapy.

## 1. Introduction

Glioblastoma (GBM) is a grade IV malignant glioma tumor that accounts for the majority (57%) of malignant glioma patients and that remains the most common cause of death due to primary malignant brain tumors in humans [1,2]. Radiotherapy, chemotherapy, and surgical debulking remain the current standards of care for malignant glioma, but even tumor resection is difficult because of its location and potential neurological impairment [3]. GBM has one of the worst prognoses with a median survival of around 15 months [3,4]. Its highly aggressive nature, molecular heterogeneity, the ability of resistant cancer stem cells to regrow post-therapy, the invasion of critical regions of the brain, and the inadequacy of achieving high therapeutic levels of chemotherapeutics in the brain because of the blood–brain barrier (BBB) are some of the key factors that constitute the vast amount of unmet need in GBM patients (as reviewed in [5]).

The recent success of immunotherapy in clinic, especially with immune checkpoint inhibitors (ICIs) that impede the engagement of programmed cell death 1 (PD-1) or cytotoxic T lymphocyte-associated protein 4 (CTLA-4) with their respective ligand or receptor to boost anti-cancer immunity, has paved a way for these agents to become a part of standard treatment in many cancer types. ICIs have been shown to be effective in patients with an increasingly wide variety of tumors [6]; however, the magnitude and duration of response to ICIs in solid tumors remains greatly variable. Although some of the hallmarks of cancer such as the degree of mutational burden [7], defective DNA-repair mechanisms [8], and checkpoint ligand expression [9] have been helpful in predicting the potential efficacy of ICIs, but the accuracy in predicting individual ICI responders using these hallmarks remains limited, owing to the complexity of interactions between cancer cells and the immune system [10]. Chimeric antigen receptor (CAR) T cell therapy is another option that appears promising for treating malignant glioma; however, high molecular heterogeneity can lead to moderation of the response to the treatment, similar to cancer vaccines, since both of these therapies are dependent on the expression of specific antigen molecules by tumor cells.

Oncolytic virotherapy uses replication-competent viruses that can selectively replicate and kill cancer cells [11,12,13,14,15,16]. Oncolytic viruses (OVs) lead to cancer cell death through different mechanisms including apoptosis, pyroptosis, and necroptosis. Direct oncolysis releases a wide range of tumor-associated antigens (TAAs)/neoantigens or danger-associated molecular patterns and viral pathogen-associated molecular patterns, which trigger inflammatory immune responses in the tumor microenvironment (TME) [17]. A highly immunosuppressive TME is a characteristic of malignant glioma and other tumor types when they metastasize into the central nervous system (CNS) compartment. The local immunosuppression in and around malignant glioma tumors is due to the deletion and development of T cell tolerance against tumor-specific antigens, in conjunction with systemic immunosuppression due to the sequestration of T cells in the bone marrow [18,19]. OVs can increase immune cell infiltration and trigger inflammation within the TME, which could be crucial in breaking the immune tolerance and can improve tumor responsiveness to ICIs [20]. A wide range of OVs are being tested both at the preclinical and clinical level in malignant glioma. An increasing number of OVs are in various phases of clinical trials, amongst which some promising OV candidates are adenovirus (DNX-2401), poliovirus (PVS-RIPO), and retroviral vector (Toca 511), which induced a durable response in 20% of malignant glioma patients and has been put on a fast track to be reviewed by the US Food and Drug Administration (FDA) [21,22,23]. Furthermore, an application for oncolytic herpesvirus G47Δ has been submitted to Japan’s Ministry of Health, Labour, and Welfare (MHLW) for treatment of patients with malignant glioma [24]. This is the first instance that an application for an OV to treat malignant glioma has been filed to a regulatory authority in any country.

## 2. Current Treatment Options for Malignant Glioma

### 2.1. Standard Therapy: Surgery and Chemoradiation

Surgical abscission remains at the core of treatment for malignant glioma along with adjuvant chemoradiation [3]. Temozolomide (TMZ) is an FDA-approved chemotherapy agent for malignant glioma and is administered concomitantly with radiation as well as an adjuvant therapy [3]. However, TMZ has been linked with increasing mutation rates resulting in defective DNA repair mechanisms that can lead to the development of resistant malignant glioma cell subpopulations, making the drug ineffective in previously responsive patients [25]. Poor overall survival with the existing standard treatments and the emergence of resistant phenotypes has created an urgent need for newer therapeutics in malignant glioma patients. Several new therapies such as oncolytic virotherapy, immunotherapy, CAR T cell therapy, and cancer vaccines are currently under investigation in preclinical and clinical studies, and their approval for clinical application in malignant glioma patients is awaited.

### 2.2. Immunotherapy

A targeted treatment drug, Bevacizumab, has also been approved by the FDA for treating recurrent malignant glioma patients [26]. Bevacizumab is a monoclonal antibody directed against vascular endothelial growth factor that acts by limiting angiogenesis in malignant glioma tumors restricting tumor growth, but eventually it can lead to the development of a resistant phenotype because of a transition in mesenchymal gene expression [27]. The discovery of ICIs has been revolutionary, resulting in the approval of several ICIs blocking PD-1, CTLA-4, and programmed cell death receptor 1 ligand (PD-L1) to treat various cancer types. However, the application of ICIs in solid tumors is challenging, and their efficacy against GBM or brain metastases is limited [6]. Based on the response to ICIs, tumors are broadly classified as non-responding (“cold”) or responsive (“hot”) tumors [28]. Solid tumors are in a constantly transitional state with an increasing degree of heterogeneity and can develop adaptive resistance to therapies. The magnitude of innate and adaptive resistance to ICIs in tumors is the determining factor for efficacy of these therapies [29]. Among solid tumor types, malignant gliomas have been reported to have a high degree of both intrinsic and adaptive resistance to immunotherapies unlike melanoma that show a low level of both intrinsic and adaptive resistance [30,31,32]. Although the FDA has approved the PD-1 blocker, pembrolizumab for pan-cancer application in tumors with a high mutational burden, including glioma and other solid tumors, concerns have been raised for its application in malignant glioma patients because of the distinct differences in the immunological attributes like local and systemic immunosuppression between glioma and other cancer patients [33].

Understanding the underlying mechanisms behind the development of innate and adaptive resistance to ICIs can help in designing better treatment strategies where ICIs can be used in combination with other therapeutic agents in tumors that are non-responsive to ICI monotherapies. The efficacy of ICIs is dependent on the degree of expression of the target checkpoint receptors on tumor and peritumoral cells, and the heterogeneous nature of malignant glioma tumors can be a major hurdle to success. Systemic immunosuppression [19], poor immune cell infiltration of tumors, and suboptimal delivery of systemically administered ICIs due to the BBB [34] are some of the other factors that limit the efficacy of ICIs in malignant glioma. Although the list of FDA-approved agents in this category is continually expanding, application of these agents in GBM patients will require caution and significant optimization.

### 2.3. Chimeric Antigen Receptor T Cell Therapy

CAR T cell therapy uses autologous T cells engineered to target specific tumor antigens expressed on the surface of tumor cells for tumor eradication. CAR T cell therapies have produced sustained therapeutic effects in refractory hematological cancers, but their success in the treatment of solid tumors has also been limited [35,36,37]. The efficacy of CAR T cells is restricted in malignant glioma mainly because of the high degree of tumor heterogeneity, the BBB, and significantly immunosuppressive TME. Several CAR T cell therapies targeting a range of tumor antigens such as EGFR (NCT01454596, NCT03638167, NCT02844062, NCT02331693, NCT03726515), GD2 (NCT04196413, NCT04099797), HER2 (NCT03500991, NCT03389230, NCT01109095), IL13Rα2 (NCT02208362, NCT04661384, NCT04003649), B7-H3 (NCT04185038, NCT04077866, NCT04385173), and CD-147 (NCT04045847) are currently under clinical investigation in malignant glioma. The loss of target antigen expression by glioma cells renders the CAR T cell therapies ineffective, as was evident by the decrease in or loss of IL13Rα2 and epidermal growth factor receptor variant III (EGFRvIII) [35,36] antigens by tumor cells in patients with recurrent malignant glioma. CAR T cell therapies that can target multiple tumor antigens to avoid dependence on a single target antigen for efficacy or the simultaneous use of multiple CAR T cell cocktails, targeting different tumor antigens can help overcome tumor resistance due to variable antigen expression [37]. Furthermore, hypoxic environment in malignant glioma tumors has been linked to an increase in expression of hypoxia response elements, which induce higher levels of PD-L1 expression in the TME, thus leading to the suppression of T cell responses [38,39]. High levels of immune-suppressive factors like transforming growth factor (TGF)-β, interleukin (IL)-4, IL-10, Arg1, IDO, and PD-L1 produced by tumor-associated myeloid derived suppressor cells (MDSC), regulatory T (Treg) cells, and tumor-associated macrophages/microglia (TAMs) further contribute to the subdued immune response in malignant glioma [40].

Immunomodulation in malignant glioma tumors blocks the activation of immune response pathways important for successful CAR T cell therapy and leads to T cell exhaustion. Therefore, CAR T cell therapy needs further optimization in malignant glioma with improved (1) accessibility to the brain; (2) tumor cell targeting; (3) survivability in immunosuppressive TME; and (4) the ability to proliferate and exert therapeutic effects with minimal immune-based toxicities [41].

### 2.4. Vaccines

There are two major types of vaccines that are under investigation for malignant glioma therapy, peptide vaccines, and dendritic-cell-based (DC) vaccines. Peptide vaccines use small tumor-specific antigen sequences up to 30 bases to induce anti-tumor immune responses. Several vaccines targeting single or multiple tumor antigens are under investigation and have shown some encouraging results in malignant glioma [42,43,44]. EGFRvIII is overexpressed in malignant gliomas. An EGFRvIII peptide vaccine, Rindopepimut, was tested in a phase II trial in newly diagnosed EGFRvIII-expressing GBM patients. The vaccine induced anti-EGFRvIII antibodies and resulted in 66% progression-free survival (PFS) at 5.5 months, but 67% of tumor samples collected after more than 3 months of treatment showed loss of EGFRvIII expression [45]. Similar results were reported in a phase III study of Rindopepimut in GBM patients where the vaccine improved PFS, but 82% of tumor samples (*n* = 11) from patients with recurrent disease showed loss of EGFRvIII expression [46]. Unfortunately, Rindopepimut failed to improve the survival in newly diagnosed EGFRvIII positive GBM patients in combination with temozolomide in a phase III trial where patients showed a median overall survival of 20.1 months against 20 months in the control group receiving temozolomide [31]. Another peptide vaccine, IMA950, which is a multi-peptide vaccine, was well tolerated and the primary immunogenicity endpoint against tumor-associated antigens was exceeded in at least 30% of patients in combination with granulocyte monocyte-colony stimulating factor (GM-CSF) in newly diagnosed GBM patients [47]. However, IMA950 in combination with poly-ICLC, a synthetic toll-like receptor 3 ligand, showed no improvement in PFS and overall survival in high-grade glioma patients [48]. Among two other vaccines, isocitrate dehydrogenase 1 (IDH1) met the safety end point in a phase I study [49] and autologous heat-shock protein vaccine in combination with standard therapy improved overall survival in a phase II study in GBM patients [50]. Initial clinical studies with both these vaccines appear encouraging and warrant further investigation.

DC vaccines are based on exposing autologous DCs to tumor antigens ex vivo and administrating the activated DCs into patients. Several clinical studies are currently testing DC vaccine therapies in glioma patients (NCT02649582, NCT02709616, NCT01567202, NCT02772094, NCT02366728, NCT02465268, NCT01204684, NCT02754362, NCT03395587, NCT03400917). DCVax-L uses autologous phagocytic DCs exposed to immunologically enhanced glioma cells by interferon (IFN)-γ and heat-shock treatment, derived from patients, instead of using single or limited tumor antigens, which help DCVax-L to expand its targeting potential. DCVax-L showed an improved median survival in grade 4 glioma patients in a phase I/II study [51]. Additional clinical trials are currently underway to evaluate DCVax-L in glioma patients (NCT03014804, NCT00045968).

Considering the molecular heterogeneity in malignant glioma tumors, both peptide- and DC-based vaccines targeting a single tumor antigen are likely to mediate transient effects but ultimately will lead to recurrent disease because of antigen escape and regrowth of tumor cells lacking the target antigen expression. DCVax-L attempts to compensate for this heterogeneity by exposing DCs to tumor cell lysates instead of specific tumor antigens but poses a risk of inducing an autoimmune reaction. Local and systemic immune suppression in glioma patients continues to be an obstacle in executing immune-cell-mediated effects of therapeutics like vaccines. Although clinical studies have provided us with evidence that both peptide- and DC-based vaccines have the potential to induce anti-tumor immune responses, vaccines will need to overcome the major hurdle of heterogeneity in glioma tumors to exert sustained efficacy and lower the recurrence rate. The immune stimulatory potential of vaccines can, however, be exploited in combination with other therapies to achieve a synergistic effect.

## 3. Oncolytic Virotherapy for Malignant Glioma

Oncolytic virotherapy faces a unique set of challenges associated with malignant glioma, due to several roadblocks, including the BBB between vascular and CNS compartments, the tumor-protective immune environment, and a high variability in molecular attributes of tumor cells, which is discussed in detail later in this review. Most OVs, if not all, that are in clinical trials in malignant glioma patients are being delivered locally to achieve an effective virus load in the tumors. At present, herpesvirus, adenovirus, vaccinia virus, reovirus, parvovirus, poliovirus, measles virus, replicating retrovirus vector, and Newcastle disease virus (NDV) are being tested in malignant glioma patients for safety and efficacy at different clinical phases (Table 1). Although most OV candidates being tested in glioma patients have been genetically engineered to improve their safety, a select few have been designed to express therapeutic immune-stimulatory proteins. OVs engineered to express immune-stimulatory proteins not only disrupt the immunosuppressive TME but can also recruit, activate, and promote pro-inflammatory immune cells at the tumor site. Engineering OVs to deliver a payload of therapeutic proteins at tumor sites has become a well-recognized strategy to optimize therapeutic efficacy, while minimizing the systemic toxicity afforded by these therapeutic proteins.

### 3.1. Oncolytic Herpesvirus

Conditionally replicating herpes simplex virus (HSV)-1 derivative, G207, contains deletions in the *γ_1_34.5* and *ICP6*/*UL39* genes that prevent virus killing of normal brain cells. The safety of this virus was demonstrated following stereotactic inoculation of enhancing/actively growing sites of recurrent malignant gliomas and subsequent inoculation into the tumor bed cavity following tumor resection in phase I/II (NCT00028158) and phase Ib [52] studies. No toxicity or adverse events related to the virus were reported [52,53]. Results of phase II studies are not available yet. The University of Alabama at Birmingham tested the safety of G207 in multiple phase I studies, both as a monotherapy and in combination with radiation in recurrent gliomas in adults. Even with a high dose of 3 × 10^9^ plaque forming units (pfu), no virus-related toxicities were reported [53,54]. Another phase I study is testing the safety of G207 by itself or in combination with radiation in pediatric brain tumors, including malignant glioma (NCT02457845). Additionally, a phase II trial with G207 is testing the efficacy of the virus alone or the virus combined with a single low dose of radiation in pediatric patients with recurrent or progressive high-grade glioma (NCT04482933).

G47Δ is a triple-mutated, third-generation oncolytic HSV-1, generated by introducing an additional genetic mutation in the viral genome of second-generation HSV-1, G207 [55]. This virus is being investigated in several tumor types, including malignant glioma. G47Δ received designation as a breakthrough drug for treatment of malignant glioma by the MHLW, Japan, allowing its priority review for expedited approval by the Pharmaceuticals and Medical Devices Agency of Japan (PMDA) earlier in 2016 [56]. A phase I-IIa study in Japan tested G47Δ safety in patients with progressive GBM, which showed that patients tolerated the virotherapy well with no toxicity (UMIN000002661, Japan) [57].

**Table 1:** Oncolytic viruses in clinical trials for treatment of malignant glioma.

Virus		Modification	Phase	Status	Reference	Route of Delivery	Results
HSV-1	G207	Deletions at both *γ_1_34.5* and *ICP6* genes	I & II	Completed	NCT00028158 [53]	i.t./tumor resection cavity	No toxicity or serious adverse events.
			Ib	Completed	[52]	i.t.	No neurological adverse events after multiple virus dosages.
			I	Active, not recruiting	NCT02457845	i.t.	
			II	Not yet recruiting	NCT04482933	i.t.	
	G47Δ	G207 with triple mutations	I-IIa	Completed	UMIN000002661 (Japan)	i.t.	No toxicity or serious adverse events.
			II	Ongoing	UMIN000015995 (Japan)	i.t.	No toxicity with 1-year survival rate of 92.3% in 13 patients.
	rQNestin34.5v.2	Glioma-selective transcriptional regulator for expression of *ICP34.5*	I	Recruiting	NCT03152318	i.t.	
HSV-1	M032	Deletions at both *γ34.5*; expression of human *IL-12*	I	Recruiting	NCT02062827	i.t.	
	C134	Deletions at both *γ34.5*; Expression of HMCV *IRS1* gene	I	Active, not recruiting	NCT03657576	i.t.	
	HSV-1716	Deletion of both copies of *RL1*-gene-encoding ICP34.5 protein	I	Completed	[58]	i.t.	No adverse effects with 4 out of 9 patients surviving 14–24 months after virotherapy.
			I	Completed	[59]	Tumor resection cavity	No toxicity with 3 out of 12 patients surviving over a year.
			I	Terminated	NCT02031965	i.t.	NA
Adeno-virus	DNX-2401	Deletion of 24 base pairs from *E1A;* expression of RGD peptide motif	I	Completed	NCT00805376 [22]	i.t.	No dose-limiting virus toxicities reported with enhanced long-term survival and T cell response to tumors.
			I	Active, not recruiting	NCT03178032	Tumor resection cavity	
Adeno-virus	DNX-2401	Deletion of 24 base pairs from *E1A;* Expression of arginine-glycine-aspartate peptide motif BM-hMSCs loaded with the DNX-2401DNX-2401 + Pembrolizumab	I	Completed	NCT01582516	i.t.	NA
			I	Completed	NCT01956734	i.t.	NA
			I	Completed	NCT02197169	i.t.	NA
			I	Recruiting	NCT03896568	i.a.	
			II	Active, not recruiting	NCT02798406	i.t.	
	DNX-2440	DNX-2401 expressing OX40L	I	Recruiting	NCT03714334	i.t.	
	NSC-CRAd-Survivin-pk7	*E1A* expression under the control of human *Survivin* promoter; NSCs loaded with CRAd-survivin-pk7	I	Active, not recruiting	NCT03072134	i.t.	
	ONYX-015	E1B-attenuated adenovirus	I	Completed	[60]	Tumor resection cavity	No serious adverse effects with 10^10^ pfu of virus; among 24 patients, 1 patient each showed no progression and regression.
Vaccinia	TG6002	Deletions of *TK* and *14L;* expression of transgene *FCU1*	I/II	Recruiting	NCT03294486	i.v.	
Reovirus	Reolysin	None	I	Completed	NCT00528684	i.t.	No dose-limiting toxicity even with the highest does of 1X10^10^ TCID_50_.
			I	Completed	[61]	i.t.	No high-grade adverse effects. One and 10 out of 12 patients had a stable and progressive disease, respectively.
			Ib	Completed	[62]	i.v.	Reovirus is capable of infecting glioma tumors when injected i.v. and increases cytotoxic T cell infiltration in tumors.
		Reovirus + Sargramostim	I	Active, not recruiting	NCT02444546	i.v.	
Parvovirus H-1	H-1PV		I/II	Completed	NCT01301430 [63]	i.v. or i.t. + tumor resection cavity	Virus was safe and well-tolerated. Induced cytotoxic T cell response.
			I/IIa	Completed	[64]	i.t./i.v.	Enhanced immune response and improved median survival.
Poliovirus	PVSRIPO	Poliovirus IRES switched with HRV2 IRES	I	Recruiting	NCT03043391	i.t.	
			I	Active, not recruiting	NCT01491893	i.t.	Improved survival rate with no neurovirulence.
			II	Active, not recruiting	NCT02986178	i.t.	
Measles Virus	MV-CEA	Measles virus expressing CEA	I	Completed	NCT00390299	Tumor resection cavity	NA
Retroviral vector	Toca511	Replicating retroviral vector expressing cytosine deaminase	I	Completed	NCT01470794	Tumor resection cavity	Durable response rate in subgroup of malignant glioma patients.
			I	Completed	NCT01156584	i.t/i.v.	NA
			II & III	Terminated	NCT02414165	Tumor resection cavity	Failed to improve survival and meet other efficacy endpoints.
Newcastle disease virus	NDV-HUJ strain	Mutation at F1-F2 junction	I/II	Completed	[65]	i.v.	No severe toxicity with complete remission in 1 patient.
	MTH-68/H		I	Completed	[66]	i.v.	No adverse effects with improved survival of 4–9 years in 4 patients.

Abbreviations: IL-12, interleukin-12; HMCV, human cytomegalovirus; HRV2, human rhinovirus type 2; IRES, internal ribosome entry site.; CEA, carcinoembryonic antigen; i.t., intratumoral; i.v., intravenous; i.t.; NA, not available.

A phase II study tested the efficacy of G47Δ in malignant glioma patients, including GBM (UMIN000015995, Japan), using a dose of 1 × 10^9^ pfu, injected stereotactically into the tumor at different coordinates twice within two weeks and every four weeks thereafter, with a maximum of six doses. The treatment was well tolerated, and the interim analysis of results of this study showed a significantly higher 1-year survival rate of 92.3% in 13 patients as compared to the 15% in the control group, based on a meta-analysis of historical data. The high efficacy of G47Δ in this phase II study led to early termination of the trial as the evidence from the study was enough to submit a new drug application [67]. Based on the results of a phase II study, a new drug application for G47Δ has recently been submitted for treating patients with malignant glioma to Japan’s MHLW [24]. This is the only OV in any country that has reached this stage for treating malignant glioma and could well be the first OV to be approved for CNS tumor therapy.

The rQNestin34.5v.2 virus is an oncolytic HSV-1, attenuated via a glioma-selective transcriptional regulator that controls expression of the neurovirulent *ICP34.5* gene, allowing selective replication of the virus in glioma cells [68]. This virus is now in a phase I clinical trial in malignant glioma patients to assess safety when delivered intracranially (NCT03152318). The M032 is a conditionally replicating HSV-1 engineered to express interleukin (IL)-12 to boost the immune responses against tumors. It is currently being tested in a phase I trial (NCT02062827). The C134 virus is a chimeric HSV-1 variant with a deleted *γ_1_34.5* gene and expresses the human cytomegalovirus *IRS1* gene. The C134 virus is a replication-competent virus that can infect and kill tumor cells and induce an anti-tumor immune response facilitated by the *IRS1* transgene which helps in evading PKR-mediated protein shutoff [69]. A phase I study is testing the safety of C134 in CNS tumors, including malignant glioma where the virus will be delivered into tumors (NCT03657576). Another oncolytic HSV-1, HSV-1716 was tested in two phase I clinical trials where the virus was delivered intracranially. Both trials showed no virus-related toxicity with a dose as high as 1 × 10^5^ pfu and improved overall survival [58,59]. However, a newer phase I study with HSV-1716 in younger patients with refractory or recurrent high-grade gliomas has been terminated for unknown reasons (NCT02031965).

### 3.2. Oncolytic Adenovirus

Oncolytic adenovirus DNX-2401 was generated by deleting 24 base pairs from the *E1A* gene and modifying it to express arginine-glycine-aspartate (RGD) peptide in the fiber knob receptor of adenovirus type 5. The *E1A* binds to the retinoblastoma (Rb) protein, and deletion of the Rb-binding region of the *E1A* gene is expected to allow selective replication of DNX-2401 in cancer cells with disrupted *Rb* gene expression [70], while the RGD peptide motif facilitates virus entry via integrins [71]. A phase I study tested the safety and maximum tolerated dose (MTD) when injected into recurrent malignant glioma tumors and surrounding brain tissue (NCT00805376). No dose-limiting toxicities were observed in this study with the highest dose of 3 × 10^10^ virus particles (vps), and treatment increased cytotoxic T cell infiltration of tumors with an improved long-term survival [22]. Another phase I study tested tolerance of DNX-2401, following injection of 3 × 10^10^ vps into brain parenchyma along with TMZ in malignant glioma patients but has not yet published the results (NCT01956734). A combination of DNX-2401 with IFN-γ has also been studied in a phase I study in patients with recurrent malignant glioma, where virus was injected directly into tumors. Results of this study have not been published yet (NCT02197169). The MTD and toxicity of allogeneic bone-marrow-derived human mesenchymal stem cells (BM-hMSCs) loaded with the DNX-2401, when injected intraarterially, will be investigated in an upcoming phase I trial. The homing and the ability of BM-hMSCs to deliver DNX-2401 will be tested in patients with recurrent high-grade glioma (NCT03896568). The DNX-2401 is also being tested for efficacy in combination with pembrolizumab, an ICI in a phase II study in recurrent malignant glioma, where a single dose of virus ranging from 5 × 10^8^ to 5 × 10^10^ will be delivered intratumorally followed by intravenous (i.v.) pembrolizumab every 3 weeks for up to 2 years or until disease progression (NCT02798406).

Oncolytic adenovirus DNX-2440 is an engineered version of DNX-2401 that expresses OX40 ligand (OX40L) for stimulation of T cell responses in tumors. This virus is being tested in a phase I study among patients with recurrent malignant glioma where the virus will be delivered stereotactically (NCT03714334). Oncolytic adenovirus type 5, CRAd-Survivin-pk7, was generated by incorporating the survivin promoter to drive *E1A* gene expression and modification of the fiber protein to contain a poly-lysine (pk7) for enhancing the virus tropism in malignant glioma cells [72]. The MTD of CRAd-survivin-pk7 loaded onto neural stem cells (NSCs) will be determined in a phase I study in newly diagnosed malignant glioma patients. Patients will receive the NSC-CRAd-survivin-pk7 stereotactically along with chemoradiation (NCT03072134).

An *E1B* attenuated adenovirus, ONYX-015, has also been tested in a phase I study in patients with recurrent glioma and showed no serious virus-associated adverse effects with a dose as high as 1 × 10^10^ pfu. Among 24 patients in the study, one each showed non-progression and regression of disease. Two patients who underwent a second resection 3 months after virus injection showed immune cell infiltration in the region [60].

### 3.3. Oncolytic Vaccinia Virus

Oncolytic vaccinia virus TG6002 is an attenuated virus engineered to express the yeast *FCU1* gene, which encodes cytosine deaminase and uracil phosphoribosyl transferase, allowing the local transformation of the pro-drug flucytosine (5-FC) into cytotoxic 5-fluorouracil (5-FU) and 5-fluoro-uridilyl monophosphate at targeted sites by the virus, such as tumors. Combination of TG6002 with 5-FC showed tumor-selective viral replication, prolonged maintenance of therapeutic levels of 5-FU in tumors, and significant antitumor effects in multiple human xenograft tumor models [73]. TG6002 with 5-FC is currently being tested in patients with recurrent malignant glioma (NCT03294486). The phase I portion of the study will determine the MTD for TG6002, defining an appropriate dose of TG6002 for combination with 5-FC in the phase II study. The virus will be injected i.v. in these studies.

### 3.4. Oncolytic Reovirus

A phase I study to determine the MTD, the dose limiting toxicity (DLT), and the anti-tumor effects of REOLYSIN, a therapeutic reovirus, in patients with malignant glioma when administered intralesionally has been completed (NCT00528684). Reovirus dose ranges from 1 × 10^8^ to 1 × 10^10^ TCID_50_ were tested in this phase I study, and the highest dose tested will be used in the phase II study. The phase I study could not identify that the DLT and MTD dose was not reached; however, there was evidence of antitumor activity in some patients. This is the first report that demonstrated the safety and tolerance of intratumoral infusion of reovirus in patients with recurrent malignant glioma [74]. The safety of oncolytic reovirus was also demonstrated in another phase I study, where no level 3 or 4 adverse effects due to treatment were observed with local administration of the virus [61]. A subsequent phase Ib study showed that the reovirus is capable of reaching and infecting glioma tumors when injected i.v. and enhanced leukocyte infiltration into tumors [62].

### 3.5. Oncolytic Parvovirus

Two studies have tested oncolytic parvovirus in malignant glioma patients in a clinical set up. In a phase I/II study, the first dose of parvovirus H-1 was delivered intratumorally or intravenously, and the second dose was administered after surgical removal of the tumor around the resection cavity after 10 days (NCT01301430). The treatment was safe and well tolerated by patients. Of note, the virus also demonstrated the ability to cross the BBB and infect tumors to trigger cytotoxic T cell responses [63]. In another phase I/IIa study, systemically administered oncolytic parvovirus was able to infect malignant glioma tumors and enhance recruitment of activated cytotoxic T lymphocytes and TAMs in malignant glioma patients [64]. These studies have demonstrated the ability of parvovirus to induce an immune response in immunosuppressive glioma tumors, even when administered systemically.

### 3.6. Oncolytic Poliovirus

Oncolytic poliovirus PVSRIPO has been generated by switching the original internal ribosome entry site (IRES) with the IRES from human rhinovirus 2 (HRV2). PVSRIPO has demonstrated excellent safety and efficacy in a wide range of tumor types, including malignant glioma [75,76,77,78,79,80,81]. The virus was tested in a phase I study where intratumoral infusion of PVSRIPO in patients with recurrent grade IV malignant glioma showed no neurovirulence, and the survival rate among patients was higher at 24 and 36 months as compared to historical controls (NCT01491893). PVSRIPO is one of the only two oncolytic viruses along with oncolytic HSV-1, HSV-1716, which is presently being tested in a phase I study to determine the safety and potential toxicity in young populations between 12–21 years of age with malignant glioma. In this phase I study, PVSRIPO will be delivered as a single intratumoral dose using an intracerebral catheter (NCT03043391). Another phase II study is testing PVSRIPO in grade IV malignant glioma patients for safety and efficacy, where patients will be administered PVSRIPO intratumorally via convection-enhanced delivery (CED) in the enhancing portion of the tumor (NCT02986178).

### 3.7. Oncolytic Measles Virus

An engineered measles virus expressing carcinoembryonic antigen (MV-CEA) is being investigated in recurrent malignant glioma patients where the virus was delivered intratumorally or in the tumor bed. The study focused on the safety, toxicity, and MTD but also assessed efficacy in a preliminary manner (NCT00390299). The first group of patients in this study received direct MV-CEA escalating doses from 1 × 10^5^ to 2 × 10^7^ TCID_50_ injected in the excised tumor cavity. The second group of patients received the MV-CEA after the dose escalation reached 1 × 10^7^ TCID_50_ in the first group. In the second group, the first dose of MV-CEA was injected directly into recurrent tumors, followed by resection of tumors 5 days post-first virus injection, and the second dose of virus was administered into the tumor cavity. Preliminary results showed no DLT with use of intracranial MV-CEA doses as high as 2 × 10^7^ TCID_50_ (as reviewed in [82]).

### 3.8. Oncolytic Retrovial Vector Toca511

Vocimagene amiretrorepvec (Toca 511) is a gamma-retroviral replicating vector that encodes cytosine deaminase that converts prodrug 5-FC (Toca FC) to 5-FU in rapidly dividing cells, leading to targeted effects of the chemotherapeutic 5-FU. A phase I study with Toca511 administered with Toca FC (NCT01470794) showed a durable response rate in a subgroup which included both IDH-1 mutant and wild-type glioma tumors. The Toca511 and Toca FC combination is also being investigated in recurrent malignant glioma patients in another phase I study (NCT01156584), but the results are not yet available. A recent phase III study of Toca511+Toca FC combination (NCT02414165) has been terminated, since it failed to demonstrate improvement in survival or meet any other efficacy endpoints among patients with high-grade glioma [83].

### 3.9. Oncolytic Newcastle Disease Virus

There are two NDV strains that are currently being investigated in clinical studies for glioma treatment. NDV-HUJ is an attenuated strain with mutation in the cleavage site between fusion proteins F1 and F2, whereas MTH-68/H is a pathogenic strain that differs in amino acid sequence at the F1-F2 junction from NDV-HUJ [84]. A phase I study showed that patients with recurrent malignant glioma tolerated i.v. injection of oncolytic NDV-HUJ and had minimal toxicity. One patient among 11 total who received the treatment achieved a complete response [65]. The pathogenic NDV stain, MTH-68/H, resulted in increased survival time up to 5–9 years in four patients with high-grade glioma, which was higher than the expected survival and enhanced the quality of life. These patients received only MTH-68/H as a non-surgical onco-therapy [66].

## 4. Challenges in Treating Malignant Glioma with Oncolytic Virus

### 4.1. Getting Beyond the Blood–Brain Barrier

**Figure 1:**
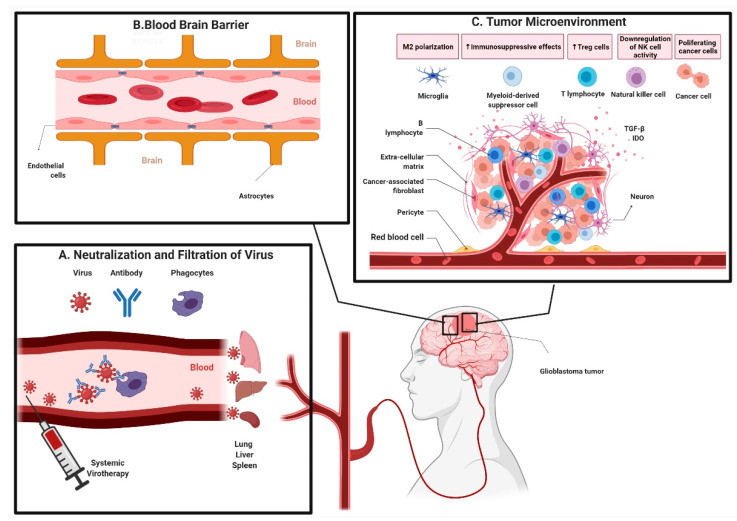
Overview of hurdles to oncolytic virotherapy in malignant glioma. (**A**) Neutralization and non-specific filtration of virus. Systemically administered oncolytic viruses are prone to complement and antibody-mediated neutralization and uptake by phagocytic macrophages. Virus particles are further non-specifically filtered in lungs, liver, spleen, and other tissues as they pass through them reducing the overall number of virus particles that reach tumors. (**B**) Blood–brain barrier. Transportation across blood vessels into the brain tissue is tightly regulated by specialized perivascular architecture of cells, known as the blood–brain barrier, which also hinders transport of oncolytic viruses to tumors within the central nervous system compartment, thereby reducing the virus load delivered into tumors. (**C**) Immunosuppressive tumor microenvironment. Immunosuppressive cells like M2 phenotype microglia/macrophages, myeloid-derived suppressor cells, regulatory T cells are upregulated and maintained because of high levels of tumor necrosis factor (TGF)-β and indoleamine 2,3-dioxygenase (IDO) expressed by tumor cells, which form a tumor-protective surrounding around a tumor (This is an original figure created with biorender.com (accessed on 30 April 2021)).

Upon systemic delivery, OVs have to face several obstacles before reaching the tumors, including neutralization by complement factors and/or antibodies and anti-viral immune cell responses. Moreover, non-specific virus uptake in tissues such as liver, spleen, lung, and tissue resident macrophages further reduce the viral load that can reach tumors (Figure 1A) [85]. Furthermore, an inefficient extravasation of virus from vascular to extravascular compartments due to physical barriers curtails the virus particles reaching tumors. The physical BBB in the CNS-regulating passage of virus from vascular to extravascular compartments is even more stringent (Figure 1B). The architecture of microvasculature in the CNS is unique where different cell types such as endothelial cells, pericytes, microglia, and astrocytes form a complexly interactive system. The continuous non-fenestrated blood vessels in the BBB tightly regulate transport of molecules, ions, and cells across the blood vessel membrane to the brain, which is critical for maintenance of homeostasis and optimal functioning of neurons. Additionally, the BBB plays a critical role in protecting the brain from inflammation, toxins, and injury (as reviewed in [34]). The BBB, however, is a major obstacle in delivering systemic therapeutics to tumors located in the CNS compartment, including OVs [34].

Despite all the hurdles associated with the systemic delivery of OVs, some viruses have shown the ability to effectively cross the BBB to reach and infect tumors in animal models, such as Semliki Forest virus [86], vaccinia virus [87,88], chimeric vesicular stomatitis virus (VSV) [89], parvovirus H-1 [90], Mengovirus [91], and Seneca Valley virus-001 [92] when administered systemically. Oncolytic parvovirus H-1 [63] has also been shown to reach malignant glioma tumors when delivered systemically in glioma patients. Oncolytic reovirus is another virus that has been shown to reach brain tumors when injected systemically in both animal models and patients. Reovirus is thought to be carried by immune cells across the BBB [62].

Most, if not all, OVs that are in clinical trials for treating malignant glioma are being administered locally to circumvent the barriers associated with systemic delivery of viruses and maximize the virus load in tumors for optimum efficacy. The OVs that are being injected systemically in glioma patients in the ongoing or completed trials include vaccinia virus (NCT03294486), reovirus [62], parvovirus [63,64] (NCT01301430), NDV [65] and adenovirus (NCT03896568). The adenovirus, however, is being loaded on carrier cells before systemic administration. Tumor tropism of neural and mesenchymal stem cells can be exploited by using them as carriers for OVs. Several preclinical studies have demonstrated that OVs loaded on stem cell carriers can be effectively delivered to malignant glioma tumors when injected systemically (as reviewed in [93]). The use of carrier stem cells as “trojan horses” can effectively deliver OVs that are restricted by the BBB when injected systemically. Currently, two clinical trials are investigating this strategy to deliver the virus to tumors in malignant glioma patients. As described earlier, allogeneic BM-hMSCs and NSCs are being used to carry the oncolytic adenoviruses, DNX-2401 (NCT03896568) and CRAd-Survivin-pk7 (NCT03072134) in two different clinical trials in malignant glioma patients. BM-hMSCs are known to show natural tropism for human gliomas, which facilitates the homing of DNX-2401-loaded BM-hMSCs to glioma tumors [94]. Furthermore, this study used the endovascular selective intra-arterial administration (ESIA) technique that facilitates localized delivery of BM-hMSCs to glioma tumors by disrupting the BBB using hyperosmotic solution (as reviewed in [95,96]). NSCs loaded with CRAd-survivin-pk7 will be delivered directly into tumors with the goal of enhancing virus spread within the tumor. Furthermore, CED is a minimally invasive technique that establishes a pressure gradient using a catheter to locally deliver therapeutics in brain. CED helps to maximize the uptake of therapeutic agents by tumor cells, bypassing the BBB [97]. A phase II study showed that the intratumoral CED of oncolytic poliovirus, PVSRIPO, improved the overall survival in malignant glioma patients [21].

To summarize, some of the OVs have a natural tropism for neuronal tissue or naturally use immune cells as carriers, enabling them to cross the BBB to infect and kill tumor cells in the CNS compartment; however, most OVs have difficulty in crossing the BBB, upon systemic delivery. The BBB poses as a major hurdle in ensuring efficacious levels of therapeutics are achieved in malignant glioma tumors, including OVs. Carrier cells offer a promising alternative that can help improve the delivery of OVs across the BBB, while ESIA and local CED can potentially maximize the uptake of OVs by tumor cells. Together these strategies can be used to achieve therapeutic levels of OVs in CNS tumors for optimal efficacy.

### 4.2. Changing the Tumor Landscape: From Cold to Hot

There was an earlier notion that malignant glioma tumors are immunologically privileged because of the isolation from surrounding structures by the BBB [98]; however, there is growing evidence that immune cells can cross the BBB, especially in neuroinflammatory conditions. This evidence represents a window of opportunity that requires exploration as it may be exploited to potentiate immune- and virotherapies in malignant glioma [99,100]. A thorough understanding of the underlying mechanisms involved in enhanced immune cell infiltration in the CNS during neuroinflammatory conditions will be required to optimize anti-tumor immune responses in glioma patients. There is no dearth of immune cells in malignant glioma tumors but rather an abundance of immune cells with TAMs constituting up to 30–50% of the cellular mass in tumors [101]. It appears that a major part of local resistance to immunotherapeutics in malignant glioma tumors comes from their highly immunosuppressive TME (Figure 1C). The efficacy of immunotherapy is further limited in malignant glioma patients because of severe systemic immunosuppression [102].

Local immunosuppression in malignant glioma tumors is mediated through both the suppression of immune effector cells and the stimulation of immunosuppressive immune cell types. T cell dysfunction in the TME of malignant glioma is mediated via multiple mechanisms that lead to T cell senescence [103], exhaustion [104], tolerance [105] and anergy [106]. Furthermore, immunoreactive cells like cytotoxic T cells, natural killer (NK) cells, and M1 macrophages are downregulated, in addition to pacifying the functionality of antigen-presenting cells (APCs) by reduction of co-stimulatory cell surface receptors. Immunosuppressive cell phenotypes such as Treg cells, M2 macrophages/microglia, and MDSCs are promoted in the TME of malignant glioma via secretion of immunosuppressive cytokines [107,108]. The expression of TGF-β and indoleamine 2,3-dioxygenase (IDO) by malignant glioma tumors promote the recruitment, survival, and maintenance of Treg cells along with reduced activation and proliferation of cytotoxic T cells and inhibition of APCs [109,110]. The NK cell activity in the TME of malignant glioma is not only inhibited directly by tumor cells, which express an inhibitory ligand, HLA-G, that binds to NK receptors, reducing NK cytotoxicity but also via secretion of TGF-β, which downregulates NK cells [111,112]. The upregulated Treg cells in the TME can further inhibit NK cell functions like cytotoxic activity, cytokine production, proliferation, and tumor rejection [113]. Both MDSCs and M2 macrophages/microglia, also known as TAMs exert immunosuppressive effects in the TME and have been correlated with poor survival in malignant glioma patients [114,115,116,117].

Systemic immunosuppression in malignant glioma patients is due to T cell lymphopenia caused by sequestration of T cells in the bone marrow, spleen, and lymphoid organs. Loss of surface expression of S1P1 on T cells, which is critical for egress of T cells from the bone marrow or lymphoid tissues into systemic circulation, is believed to be responsible for severe T cell dysfunction in malignant glioma patients. Tumors located in the CNS, including malignant glioma, disrupt the S1P1–S1P axis gradient to trap T cells in peripheral organs, while causing the contracture of spleen and lymphoid organs [19].

As discussed earlier, oncolytic virotherapy can lead to tumor cell lysis, the release of TAAs, the disruption of the immunosuppressive TME, and the induction of innate immune responses in and around tumors. Induction and maintenance of immune responses against tumors is critical for extended therapeutic effects of OVs, post their immune clearance (as reviewed in [118,119,120,121,122]). Several OVs that are currently in clinical studies in malignant glioma patients have been shown to induce immune cell responses in tumors. Oncolytic adenovirus DNX-2401 treatment enhanced CD8^+^ and T-bet^+^ cell infiltration in tumors [22], while ONYX-015 treatment increased lymphocytic and plasmacytoid infiltration in the peritumoral region [60]. Increased cytotoxic T cell infiltration of tumors treated with oncolytic reovirus as compared to the control samples was also seen in malignant glioma patients [62]. Oncolytic parvovirus also increased the infiltration of activated cytotoxic T cells and TAMs with inducible nitric oxide synthase expression in tumors in malignant glioma patients [63,64].

As discussed before, malignant glioma tumors secrete immunosuppressive cytokines that help to maintain a tumor protective environment; hence, more immunostimulatory factors in the TME can help to shift the cytokine balance to boost and maintain anti-tumor immune responses. Oncolytic virotherapy uses two main approaches to achieve the higher levels of pro-inflammatory factors in the TME: (1) engineering OVs to express immunostimulatory proteins and (2) the administration of immune stimulators and OVs in combination. Several OVs have been designed to express immunostimulatory cytokines, such as IL-2 [123,124,125], IL-12 [126,127], IL15Rα-IL15 fusion protein [128], IL-4 [129], GM-CSF [130,131] and chemokines [132,133], which have shown promising results in pre-clinical studies in malignant glioma tumor models; however, this review focuses on OVs that are in clinical studies. At least two OVs, M032- and DNX-2440-expressing immunostimulatory proteins to potentiate anti-tumor immune responses are currently in clinical trials in malignant glioma patients. Oncolytic herpesvirus, M032 (NCT02062827) is engineered to express IL-12, a pleiotropic pro-inflammatory cytokine that plays a role in activating a diverse population of pro-inflammatory immune cells. Some of the known pro-inflammatory processes that IL-12 is involved in are (1) the differentiation of T helper 1 (Th1) cells; (2) the generation of cytotoxic T and lymphokine-activated killer cells; and (3) the augmentation of the cytotoxic activity of NK and cytotoxic T cells [134,135,136,137]. Another oncolytic adenovirus in clinical trial for treating malignant glioma, DNX-2440, is engineered to express OX40L (NCT03714334), the ligand for the T-cell activating receptor OX40 on the surface of T cells. The OX40L–OX40 interaction promotes the survival of activated T cells [138] and is critical for the development of a memory T cell response [139]. Lastly, oncolytic reovirus is being tested in combination with a recombinant GM-CSF, also known as Sargramostim, in a malignant glioma clinical trial, where the latter is expected to boost the production of blood cells and possibly promote the tumor-cell-killing effects of reovirus (NCT02444546).

The OVs expressing inflammatory cytokines or immune stimulatory factors can effectively turn tumors from “cold” to “hot,” while limiting the toxicities due to systemic administration of pro-inflammatory factors. Furthermore, cancer stem cells that are responsible for the recurrence of disease after chemo-radiation therapy can be effectively killed by OVs [140]. Oncolytic adenovirus Delta-24-RGD demonstrated the ability to infect, replicate, and kill glioma stem cells (GSC) derived from patients [141]. TAMs can engulf and trap OVs, preventing the efficient spreading of virus in and around glioma tumors [142,143]. It is important to consider the fact that viruses differ in their cellular tropism and can be restricted at cell entry or post-entry levels, which can limit infection of specific cell types in the TME [144,145]. Although most studies have associated TAMs with poor prognosis in GBM patients, macrophages can be polarized to either a pro-inflammatory M1 phenotype or an anti-inflammatory M2 phenotype. Therapy-based modulation of the TME can result in polarization of macrophages to an M1 phenotype and, in turn, can induce cytotoxic T cell responses in tumors. For example, a triple combination of anti-CTLA-4, anti-PD-1, and oHSV G47Δ expressing murine IL-12 (G47Δ-mIL12) increased the influx of macrophages and their M1 polarization in glioma mouse models [126,127,146]. Alternatively, non-replicating virus vectors can be used to deliver immune-stimulatory cytokines in and around glioma tumors to promote polarization of macrophages to M1 phenotype [147,148]. Macrophages can contribute up to 50% of tumor cell mass in glioma tumors which makes these cells an attractive therapeutic target [101]. Overall, the complex interaction between OVs and different cells in the TME needs to be better understood to optimize the virus spread in tumors to maximize therapeutic efficacy.

### 4.3. Innate Immunity and Oncolytic Viruses

Infection of host cells by virus can trigger an innate anti-viral inflammatory response, releasing a range of stimulatory cytokines like type I IFN, tumor necrosis factor (TNF), and IL-1, which can be crucial in triggering immune responses against TAAs [149]. Although tumor cells with defective IFN response pathways allow unrestricted virus replication and can be more susceptible to OVs, and infection of peri-tumoral cells by OVs can trigger an anti-viral state in the TME to limit virus spread and can negatively impact the efficacy of oncolytic virotherapy [150]. Modulation of the type I IFN response by using FDA-approved JAK/STAT pathway inhibitors, such as Ruxolitinib, has been shown to enhance the titers of oncolytic measles virus in patient-derived GBM xenografts [151].

Another study showed that oncolytic HSV in a murine malignant glioma model enhanced macrophage/microglia infiltration into tumors and polarized them to the pro-inflammatory M1 phenotype that triggered the apoptosis of virus-infected cells, limiting virus spread [152]. Microglia and TAMs can restrict the virus spread and reduce efficacy of OVs in glioma. Depleting innate immune cells like microglia and peripheral macrophages has been shown to improve the OV titers in brain tumors [142]. Combination therapies where immunomodulatory drugs like rapamycin and cyclophosphamide (CPA) can boost initial virus replication in tumor cells have been used synergistically to improve therapeutic outcomes in malignant glioma tumor models [87]. It can be advantageous to negate the innate anti-viral immune response in tumors and TME to promote viral replication, leading to direct tumor cell lysis; however, strong anti-tumor immune responses are critical for the immune-mediated tumor clearance. Setting a fine-tuned balance between the direct tumor cell lysis by OVs and the immune-mediated clearance of tumor cells is essential for the best treatment outcome. Nevertheless, application of IFN modulators with OVs in malignant glioma patients is extremely tricky because of heterogenous IFN responses within a tumor, systemic immunosuppression, and a lack of reliable translatable animal models.

### 4.4. Overcoming Immune Checkpoint Mediated Immune Resistance

Immune checkpoint receptors are critical to prevent an over-reactive T cell immune response by fine tuning the activation and maintenance of T cell responses. The binding of immune checkpoint receptors expressed on the T cell surface, like PD-1 and CTLA-4, with their ligands sends inhibitory signals to T cells, negatively modulating T cell activity [153,154]. Several cancer types, including malignant glioma have upregulated expression of immune checkpoint receptor ligands like PD-L1, which potentiates immunosuppression in the TME by modulating T cell activation. High PD-L1 expression has been linked with poor survival [155,156]. ICIs are an important milestone on the path of optimizing cancer therapy. ICIs can promote the activation and maintenance of T cell responses by blocking/interfering with the immune checkpoint inhibitory axis that constrains T cell responses. Three recent clinical trials testing ICI therapies in malignant glioma patients showed limited efficacy [157]. In the wake of established immunosuppressive TME and low T cell activity, the ICIs alone are unlikely to provide desired therapeutic benefits in malignant glioma patients.

OVs can kickstart inflammatory T cell responses in malignant glioma tumors that can be harnessed with ICIs. The combination of OVs and ICIs is a natural next step and can exert synergistic effects to optimize the therapeutic outcome in malignant glioma patients. Oncolytic adenovirus DNX-2401 in combination with pembrolizumab, an anti-PD1 antibody, is currently in a phase II clinical study (NCT02798406), where the latter is expected to boost the anti-tumor immune response initiated by DNX-2401. There are two other ongoing clinical trials testing the efficacy of nivolumab (anti PD-1 antibody) (NCT02017717, NCT02617589) in GBM patients. ICIs are being delivered systemically in malignant glioma patients in all current clinical studies and face the obstacle of the BBB, which may reduce their delivery into the CNS compartment. Preclinical studies have shown that the delivery of ICIs across the BBB can be improved by loading them onto nanoparticles resulting in improved overall survival [158,159]. In conclusion, considering the limited success of ICIs as a monotherapy in highly immunosuppressive glioma tumors, there is a strong rationale for combining ICIs with OVs to augment immunotherapeutic effects.

### 4.5. Tumor Heterogeneity and Oncolytic Viruses

Malignant glioma tumors, similar to other solid tumors, show both intra- and inter-tumoral molecular heterogeneity [160]. The level of heterogeneity is negatively correlated with the response to therapeutics among malignant glioma patients [161,162]. Pre-screening of patients for molecular patterns that are likely to be benefited by a given treatment in clinical trials is very challenging because of loco-regional heterogeneity among tumor subclones, which makes the selection of tumors for sampling difficult. Similar to other therapeutics, tumor heterogeneity also affects the outcome of oncolytic virotherapy. The differential expression of virus entry receptors among tumors has been shown to affect the ability of oncolytic reovirus [163] and adenovirus [164] to infect patient-derived malignant glioma cells. OVs that are retargeted against multiple receptors instead of being dependent upon the expression of a single entry receptor can expand their ability to infect more cells in a tumor despite the underlying heterogeneity [165]. Another study showed that patient-derived primary malignant GSCs showed differential levels of resistance to oncolytic HSV because of heterogeneity. An HSV engineered to express tumor-necrosis-factor-related apoptosis-inducing ligand (TRAIL) allowed the targeting of a broader tumor cell population by combining the effect of direct HSV-mediated oncolysis and TRAIL-mediated apoptosis induction, irrespective of the differential susceptibility of tumor cells to either modes of cell death [166]. Lastly, OVs that are engineered to exploit specific gene mutations like mutated *P53, Ras*, and *Rb* genes in tumors [165] or that use tumor-specific promoters to enhance their tumor-selectivity may result in a sub-optimal therapeutic efficacy because of the diversity in the level of expression of these specific mutated genes in tumor cells [167,168,169].

The combination of OVs with standard therapeutics like chemo- and radiotherapy can exert a synergistic effect in heterogeneous glioma tumors. Chemotherapeutics induce tumor cell death via different mechanisms including DNA cross-linking (TMZ, CPA, cisplatin, 5-FU), impairment of dsDNA break repair mechanisms (etoposide), and substitution of base pairs (5-FU). Similarly, radiotherapy leads to DNA damage in tumor cells to induce death. DNA repair mechanisms are critical for glioma cells to escape cell death induced by chemotherapeutics and radiation. Oncolytic adenovirus modulates DNA repair mechanisms in tumor cells to enhance tumor sensitization to both chemo and radiotherapies (as reviewed in [170,171]). Secondly, chemotherapeutics like TMZ, CPA, cisplatin, rapamycin, and 5-FU can lead to immunomodulation, boosting virus replication in tumors. Some chemotherapeutics have also been shown to boost anti-tumor immune responses when combined with armed OVs expressing immuno-stimulatory proteins by depleting Treg cells and MDSCs. Furthermore, OVs can induce immunogenic tumor cell death to boost anti-tumor immunity, since chemotherapeutics by themselves most likely cause non-immunogenic cell death, which is important for inducing anti-tumor immune responses. Thus, chemotherapeutics and OVs can work synergistically by negating the therapeutic limitations of each other. In some combination therapies, additive cytotoxic effects of OVs and chemotherapeutics can lead to enhanced tumor cell killing (as reviewed in [170]). The synergistic combinations between OVs and chemo-radiation therapies are also important in the context of tumor heterogeneity in GBM tumors, as it can limit the efficacy of these treatments as monotherapies.

In the present scenario, a practical way to tackle the tumor heterogeneity and maintain or improve the therapeutic outcome of OVs is to use multimodal combination therapies to achieve synergistic effects. Furthermore, continuous efforts will be needed to develop molecular markers that can be reliably used to identify potential responders to a particular treatment, including OVs. Multifaceted approaches will be crucial when engineering OVs that can exert multimodal effects leading to tumor cell killing to tackle the heterogeneity in glioma tumors.

## 5. Conclusions

New treatment options will be critical for improving the formidable situation of poor survivability with standard treatment options in malignant glioma patients [3,4]. Approval of oncolytic herpesvirus, T-vec, for melanoma treatment has catapulted several experimental virotherapies into clinical studies for various tumor types, including malignant glioma [172,173]. Results from some of the clinical trials testing OVs in malignant glioma patients found evidence that viruses are capable of infecting tumor cells and enhancing immune cell recruitment, despite the immunosuppressive TME [22,62,64]. Although Toca511 therapy failed to meet the end point for survival in phase III clinical trial in malignant glioma patients (NCT02414165), the recent report from Japan about submission of a new drug application seeking an approval to use the first OV, G47Δ, for malignant glioma treatment is an important milestone in the advancement of oncolytic virotherapy for malignant glioma [24]. The phase II study with oncolytic herpesvirus, G47Δ that led to the application for a new drug for malignant glioma (UMIN000015995, Japan) used multiple stereotactic injections of the virus with a maximum of six dosages [67]. These results are undoubtedly exciting, but the economic feasibility of injecting multiple dosages of virus stereotactically remains a concern. Although some OVs have shown the ability to cross the BBB to infect malignant glioma tumors, the systemic use of OVs is still limited. GBM patients present with a unique set of problems like the BBB, the immunosuppressive TME, and a high level of molecular heterogeneity, which warrants persistent efforts to optimize oncolytic virotherapy among these patients. In the meantime, the use of OVs in synergistic combination therapies to maximize therapeutic outcome is a practical approach.

## References

[1] Louis D.N., Perry A., Reifenberger G., von Deimling A., Figarella-Branger D., Cavenee W.K., Ohgaki H., Wiestler O.D., Kleihues P., Ellison D.W. (2016). The 2016 World Health Organization Classification of Tumors of the Central Nervous System: A summary. Acta Neuropathol..

[2] Ostrom Q.T., Gittleman H., Truitt G., Boscia A., Kruchko C., Barnholtz-Sloan J.S. (2018). CBTRUS Statistical Report: Primary Brain and Other Central Nervous System Tumors Diagnosed in the United States in 2011–2015. Neuro Oncol..

[3] Stupp R., Mason W.P., van den Bent M.J., Weller M., Fisher B., Taphoorn M.J., Belanger K., Brandes A.A., Marosi C., Bogdahn U. (2005). Radiotherapy plus concomitant and adjuvant temozolomide for glioblastoma. N. Engl. J. Med..

[4] Thakkar J.P., Dolecek T.A., Horbinski C., Ostrom Q.T., Lightner D.D., Barnholtz-Sloan J.S., Villano J.L. (2014). Epidemiologic and molecular prognostic review of glioblastoma. Cancer Epidemiol. Biomark. Prev..

[5] Jackson C.M., Choi J., Lim M. (2019). Mechanisms of immunotherapy resistance: Lessons from glioblastoma. Nat. Immunol..

[6] Gong J., Chehrazi-Raffle A., Reddi S., Salgia R. (2018). Development of PD-1 and PD-L1 inhibitors as a form of cancer immunotherapy: A comprehensive review of registration trials and future considerations. J. Immunother. Cancer.

[7] Rizvi N.A., Hellmann M.D., Snyder A., Kvistborg P., Makarov V., Havel J.J., Lee W., Yuan J., Wong P., Ho T.S. (2015). Cancer immunology. Mutational landscape determines sensitivity to PD-1 blockade in non-small cell lung cancer. Science.

[8] Le D.T., Durham J.N., Smith K.N., Wang H., Bartlett B.R., Aulakh L.K., Lu S., Kemberling H., Wilt C., Luber B.S. (2017). Mismatch repair deficiency predicts response of solid tumors to PD-1 blockade. Science.

[9] Topalian S.L., Hodi F.S., Brahmer J.R., Gettinger S.N., Smith D.C., McDermott D.F., Powderly J.D., Carvajal R.D., Sosman J.A., Atkins M.B. (2012). Safety, activity, and immune correlates of anti-PD-1 antibody in cancer. N. Engl. J. Med..

[10] Gibney G.T., Weiner L.M., Atkins M.B. (2016). Predictive biomarkers for checkpoint inhibitor-based immunotherapy. Lancet Oncol..

[11] Russell S.J., Peng K.W., Bell J.C. (2012). Oncolytic virotherapy. Nat. Biotechnol..

[12] Suryawanshi Y.R., Zhang T., Essani K. (2017). Oncolytic viruses: Emerging options for the treatment of breast cancer. Med. Oncol..

[13] Pol J., Buque A., Aranda F., Bloy N., Cremer I., Eggermont A., Erbs P., Fucikova J., Galon J., Limacher J.M. (2016). Trial Watch-Oncolytic viruses and cancer therapy. OncoImmunology.

[14] Pol J.G., Levesque S., Workenhe S.T., Gujar S., Le Boeuf F., Clements D.R., Fahrner J.E., Fend L., Bell J.C., Mossman K.L. (2018). Trial Watch: Oncolytic viro-immunotherapy of hematologic and solid tumors. OncoImmunology.

[15] Zhang T., Suryawanshi Y.R., Woyczesczyk H.M., Essani K. (2017). Targeting Melanoma with Cancer-Killing Viruses. Open Virol. J..

[16] Suryawanshi Y.R., Zhang T., Razi F., Essani K. (2020). Tanapoxvirus: From discovery towards oncolytic immunovirotherapy. J. Cancer Res. Ther..

[17] Achard C., Surendran A., Wedge M.E., Ungerechts G., Bell J., Ilkow C.S. (2018). Lighting a Fire in the Tumor Microenvironment Using Oncolytic Immunotherapy. EBioMedicine.

[18] Jackson C.M., Kochel C.M., Nirschl C.J., Durham N.M., Ruzevick J., Alme A., Francica B.J., Elias J., Daniels A., Dubensky T.W. (2016). Systemic Tolerance Mediated by Melanoma Brain Tumors Is Reversible by Radiotherapy and Vaccination. Clin. Cancer Res..

[19] Chongsathidkiet P., Jackson C., Koyama S., Loebel F., Cui X., Farber S.H., Woroniecka K., Elsamadicy A.A., Dechant C.A., Kemeny H.R. (2018). Sequestration of T cells in bone marrow in the setting of glioblastoma and other intracranial tumors. Nat. Med..

[20] Chon H.J., Lee W.S., Yang H., Kong S.J., Lee N.K., Moon E.S., Choi J., Han E.C., Kim J.H., Ahn J.B. (2019). Tumor Microenvironment Remodeling by Intratumoral Oncolytic Vaccinia Virus Enhances the Efficacy of Immune-Checkpoint Blockade. Clin. Cancer Res..

[21] Desjardins A., Gromeier M., Herndon J.E., Beaubier N., Bolognesi D.P., Friedman A.H., Friedman H.S., McSherry F., Muscat A.M., Nair S. (2018). Recurrent Glioblastoma Treated with Recombinant Poliovirus. N. Engl. J. Med..

[22] Lang F.F., Conrad C., Gomez-Manzano C., Yung W.K.A., Sawaya R., Weinberg J.S., Prabhu S.S., Rao G., Fuller G.N., Aldape K.D. (2018). Phase I Study of DNX-2401 (Delta-24-RGD) Oncolytic Adenovirus: Replication and Immunotherapeutic Effects in Recurrent Malignant Glioma. J. Clin. Oncol..

[23] Cloughesy T.F., Landolfi J., Vogelbaum M.A., Ostertag D., Elder J.B., Bloomfield S., Carter B., Chen C.C., Kalkanis S.N., Kesari S. (2018). Durable complete responses in some recurrent high-grade glioma patients treated with Toca 511 + Toca FC. Neuro Oncol..

[24] Manabe S., Daiichi Sankyo Company Limited (2021). Daiichi Sankyo Submits Application for Oncolytic Virus Teserpaturev (G47∆) for Treatment of Patients with Malignant Glioma in Japan.

[25] van Thuijl H.F., Mazor T., Johnson B.E., Fouse S.D., Aihara K., Hong C., Malmstrom A., Hallbeck M., Heimans J.J., Kloezeman J.J. (2015). Evolution of DNA repair defects during malignant progression of low-grade gliomas after temozolomide treatment. Acta Neuropathol..

[26] Cohen M.H., Shen Y.L., Keegan P., Pazdur R. (2009). FDA drug approval summary: Bevacizumab (Avastin) as treatment of recurrent glioblastoma multiforme. Oncologist.

[27] Piao Y., Liang J., Holmes L., Henry V., Sulman E., de Groot J.F. (2013). Acquired resistance to anti-VEGF therapy in glioblastoma is associated with a mesenchymal transition. Clin. Cancer Res..

[28] Lim M., Xia Y., Bettegowda C., Weller M. (2018). Current state of immunotherapy for glioblastoma. Nat. Rev. Clin. Oncol..

[29] Sharma P., Hu-Lieskovan S., Wargo J.A., Ribas A. (2017). Primary, Adaptive, and Acquired Resistance to Cancer Immunotherapy. Cell.

[30] Larkin J., Chiarion-Sileni V., Gonzalez R., Grob J.J., Cowey C.L., Lao C.D., Schadendorf D., Dummer R., Smylie M., Rutkowski P. (2015). Combined Nivolumab and Ipilimumab or Monotherapy in Untreated Melanoma. N. Engl. J. Med..

[31] Weller M., Butowski N., Tran D.D., Recht L.D., Lim M., Hirte H., Ashby L., Mechtler L., Goldlust S.A., Iwamoto F. (2017). Rindopepimut with temozolomide for patients with newly diagnosed, EGFRvIII-expressing glioblastoma (ACT IV): A randomised, double-blind, international phase 3 trial. Lancet Oncol..

[32] Omuro A., Vlahovic G., Lim M., Sahebjam S., Baehring J., Cloughesy T., Voloschin A., Ramkissoon S.H., Ligon K.L., Latek R. (2018). Nivolumab with or without ipilimumab in patients with recurrent glioblastoma: Results from exploratory phase I cohorts of CheckMate 143. Neuro Oncol..

[33] Khasraw M., Walsh K.M., Heimberger A.B., Ashley D.M. (2020). What is the Burden of Proof for Tumor Mutational Burden in gliomas?. Neuro Oncol..

[34] Daneman R., Prat A. (2015). The blood-brain barrier. Cold Spring Harb. Perspect. Biol..

[35] O’Rourke D.M., Nasrallah M.P., Desai A., Melenhorst J.J., Mansfield K., Morrissette J.J.D., Martinez-Lage M., Brem S., Maloney E., Shen A. (2017). A single dose of peripherally infused EGFRvIII-directed CAR T cells mediates antigen loss and induces adaptive resistance in patients with recurrent glioblastoma. Sci. Transl. Med..

[36] Brown C.E., Alizadeh D., Starr R., Weng L., Wagner J.R., Naranjo A., Ostberg J.R., Blanchard M.S., Kilpatrick J., Simpson J. (2016). Regression of Glioblastoma after Chimeric Antigen Receptor T-Cell Therapy. N. Engl. J. Med..

[37] Fesnak A.D., June C.H., Levine B.L. (2016). Engineered T cells: The promise and challenges of cancer immunotherapy. Nat. Rev. Cancer.

[38] Barsoum I.B., Smallwood C.A., Siemens D.R., Graham C.H. (2014). A mechanism of hypoxia-mediated escape from adaptive immunity in cancer cells. Cancer Res..

[39] Fang H.Y., Hughes R., Murdoch C., Coffelt S.B., Biswas S.K., Harris A.L., Johnson R.S., Imityaz H.Z., Simon M.C., Fredlund E. (2009). Hypoxia-inducible factors 1 and 2 are important transcriptional effectors in primary macrophages experiencing hypoxia. Blood.

[40] Newick K., Moon E., Albelda S.M. (2016). Chimeric antigen receptor T-cell therapy for solid tumors. Mol. Ther. Oncolytics.

[41] Akhavan D., Alizadeh D., Wang D., Weist M.R., Shepphird J.K., Brown C.E. (2019). CAR T cells for brain tumors: Lessons learned and road ahead. Immunol. Rev..

[42] Huang J., Yu J., Tu L., Huang N., Li H., Luo Y. (2019). Isocitrate Dehydrogenase Mutations in Glioma: From Basic Discovery to Therapeutics Development. Front. Oncol..

[43] Mueller S., Taitt J.M., Villanueva-Meyer J.E., Bonner E.R., Nejo T., Lulla R.R., Goldman S., Banerjee A., Chi S.N., Whipple N.S. (2020). Mass cytometry detects H3.3K27M-specific vaccine responses in diffuse midline glioma. J. Clin. Investig..

[44] Keskin D.B., Anandappa A.J., Sun J., Tirosh I., Mathewson N.D., Li S., Oliveira G., Giobbie-Hurder A., Felt K., Gjini E. (2019). Neoantigen vaccine generates intratumoral T cell responses in phase Ib glioblastoma trial. Nature.

[45] Schuster J., Lai R.K., Recht L.D., Reardon D.A., Paleologos N.A., Groves M.D., Mrugala M.M., Jensen R., Baehring J.M., Sloan A. (2015). A phase II, multicenter trial of rindopepimut (CDX-110) in newly diagnosed glioblastoma: The ACT III study. Neuro Oncol..

[46] Sampson J.H., Heimberger A.B., Archer G.E., Aldape K.D., Friedman A.H., Friedman H.S., Gilbert M.R., Herndon J.E., McLendon R.E., Mitchell D.A. (2010). Immunologic escape after prolonged progression-free survival with epidermal growth factor receptor variant III peptide vaccination in patients with newly diagnosed glioblastoma. J. Clin. Oncol..

[47] Rampling R., Peoples S., Mulholland P.J., James A., Al-Salihi O., Twelves C.J., McBain C., Jefferies S., Jackson A., Stewart W. (2016). A Cancer Research UK First Time in Human Phase I Trial of IMA950 (Novel Multipeptide Therapeutic Vaccine) in Patients with Newly Diagnosed Glioblastoma. Clin. Cancer Res..

[48] Boydell E., Marinari E., Migliorini D., Dietrich P.Y., Patrikidou A., Dutoit V. (2019). Exploratory Study of the Effect of IMA950/Poly-ICLC Vaccination on Response to Bevacizumab in Relapsing High-Grade Glioma Patients. Cancers.

[49] Platten M., Bunse L., Wick A., Bunse T., Le Cornet L., Harting I., Sahm F., Sanghvi K., Tan C.L., Poschke I. (2021). A vaccine targeting mutant IDH1 in newly diagnosed glioma. Nature.

[50] Bloch O., Lim M., Sughrue M.E., Komotar R.J., Abrahams J.M., O’Rourke D.M., D’Ambrosio A., Bruce J.N., Parsa A.T. (2017). Autologous Heat Shock Protein Peptide Vaccination for Newly Diagnosed Glioblastoma: Impact of Peripheral PD-L1 Expression on Response to Therapy. Clin. Cancer Res..

[51] Chang C.N., Huang Y.C., Yang D.M., Kikuta K., Wei K.J., Kubota T., Yang W.K. (2011). A phase I/II clinical trial investigating the adverse and therapeutic effects of a postoperative autologous dendritic cell tumor vaccine in patients with malignant glioma. J. Clin. Neurosci..

[52] Markert J.M., Liechty P.G., Wang W., Gaston S., Braz E., Karrasch M., Nabors L.B., Markiewicz M., Lakeman A.D., Palmer C.A. (2009). Phase Ib trial of mutant herpes simplex virus G207 inoculated pre-and post-tumor resection for recurrent GBM. Mol. Ther..

[53] Markert J.M., Medlock M.D., Rabkin S.D., Gillespie G.Y., Todo T., Hunter W.D., Palmer C.A., Feigenbaum F., Tornatore C., Tufaro F. (2000). Conditionally replicating herpes simplex virus mutant, G207 for the treatment of malignant glioma: Results of a phase I trial. Gene Ther..

[54] Markert J.M., Razdan S.N., Kuo H.C., Cantor A., Knoll A., Karrasch M., Nabors L.B., Markiewicz M., Agee B.S., Coleman J.M. (2014). A phase 1 trial of oncolytic HSV-1, G207, given in combination with radiation for recurrent GBM demonstrates safety and radiographic responses. Mol. Ther..

[55] Todo T., Martuza R.L., Rabkin S.D., Johnson P.A. (2001). Oncolytic herpes simplex virus vector with enhanced MHC class I presentation and tumor cell killing. Proc. Natl. Acad. Sci. USA.

[56] Fukuhara H., Ino Y., Todo T. (2016). Oncolytic virus therapy: A new era of cancer treatment at dawn. Cancer Sci..

[57] Taguchi S., Fukuhara H., Todo T. (2019). Oncolytic virus therapy in Japan: Progress in clinical trials and future perspectives. Jpn. J. Clin. Oncol..

[58] Rampling R., Cruickshank G., Papanastassiou V., Nicoll J., Hadley D., Brennan D., Petty R., MacLean A., Harland J., McKie E. (2000). Toxicity evaluation of replication-competent herpes simplex virus (ICP 34.5 null mutant 1716) in patients with recurrent malignant glioma. Gene Ther..

[59] Harrow S., Papanastassiou V., Harland J., Mabbs R., Petty R., Fraser M., Hadley D., Patterson J., Brown S.M., Rampling R. (2004). HSV1716 injection into the brain adjacent to tumour following surgical resection of high-grade glioma: Safety data and long-term survival. Gene Ther..

[60] Chiocca E.A., Abbed K.M., Tatter S., Louis D.N., Hochberg F.H., Barker F., Kracher J., Grossman S.A., Fisher J.D., Carson K. (2004). A phase I open-label, dose-escalation, multi-institutional trial of injection with an E1B-Attenuated adenovirus, ONYX-015, into the peritumoral region of recurrent malignant gliomas, in the adjuvant setting. Mol. Ther..

[61] Forsyth P., Roldan G., George D., Wallace C., Palmer C.A., Morris D., Cairncross G., Matthews M.V., Markert J., Gillespie Y. (2008). A phase I trial of intratumoral administration of reovirus in patients with histologically confirmed recurrent malignant gliomas. Mol. Ther..

[62] Samson A., Scott K.J., Taggart D., West E.J., Wilson E., Nuovo G.J., Thomson S., Corns R., Mathew R.K., Fuller M.J. (2018). Intravenous delivery of oncolytic reovirus to brain tumor patients immunologically primes for subsequent checkpoint blockade. Sci. Transl. Med..

[63] Geletneky K., Hajda J., Angelova A.L., Leuchs B., Capper D., Bartsch A.J., Neumann J.O., Schoning T., Husing J., Beelte B. (2017). Oncolytic H-1 Parvovirus Shows Safety and Signs of Immunogenic Activity in a First Phase I/IIa Glioblastoma Trial. Mol. Ther..

[64] Angelova A.L., Barf M., Geletneky K., Unterberg A., Rommelaere J. (2017). Immunotherapeutic Potential of Oncolytic H-1 Parvovirus: Hints of Glioblastoma Microenvironment Conversion towards Immunogenicity. Viruses.

[65] Freeman A.I., Zakay-Rones Z., Gomori J.M., Linetsky E., Rasooly L., Greenbaum E., Rozenman-Yair S., Panet A., Libson E., Irving C.S. (2006). Phase I/II trial of intravenous NDV-HUJ oncolytic virus in recurrent glioblastoma multiforme. Mol. Ther..

[66] Csatary L.K., Gosztonyi G., Szeberenyi J., Fabian Z., Liszka V., Bodey B., Csatary C.M. (2004). MTH-68/H oncolytic viral treatment in human high-grade gliomas. J. Neurooncol..

[67] Todo T. Results of Phase II Clinical Trial of Oncolytic Herpes Virus G47Δ in Adult Patients with Glioblastoma. https://app.oxfordabstracts.com/events/1051/program-app/submission/178511.

[68] Chiocca E.A., Nakashima H., Kasai K., Fernandez S.A., Oglesbee M. (2020). Preclinical Toxicology of rQNestin34.5v.2: An Oncolytic Herpes Virus with Transcriptional Regulation of the ICP34.5 Neurovirulence Gene. Mol. Ther. Methods Clin. Dev..

[69] Cassady K.A. (2005). Human cytomegalovirus TRS1 and IRS1 gene products block the double-stranded-RNA-activated host protein shutoff response induced by herpes simplex virus type 1 infection. J. Virol..

[70] Fueyo J., Gomez-Manzano C., Alemany R., Lee P.S., McDonnell T.J., Mitlianga P., Shi Y.X., Levin V.A., Yung W.K., Kyritsis A.P. (2000). A mutant oncolytic adenovirus targeting the Rb pathway produces anti-glioma effect in vivo. Oncogene.

[71] Fueyo J., Alemany R., Gomez-Manzano C., Fuller G.N., Khan A., Conrad C.A., Liu T.J., Jiang H., Lemoine M.G., Suzuki K. (2003). Preclinical characterization of the antiglioma activity of a tropism-enhanced adenovirus targeted to the retinoblastoma pathway. J. Natl. Cancer Inst..

[72] Ulasov I.V., Zhu Z.B., Tyler M.A., Han Y., Rivera A.A., Khramtsov A., Curiel D.T., Lesniak M.S. (2007). Survivin-driven and fiber-modified oncolytic adenovirus exhibits potent antitumor activity in established intracranial glioma. Hum. Gene Ther..

[73] Foloppe J., Kempf J., Futin N., Kintz J., Cordier P., Pichon C., Findeli A., Vorburger F., Quemeneur E., Erbs P. (2019). The Enhanced Tumor Specificity of TG6002, an Armed Oncolytic Vaccinia Virus Deleted in Two Genes Involved in Nucleotide Metabolism. Mol. Ther. Oncolytics.

[74] Kicielinski K.P., Chiocca E.A., Yu J.S., Gill G.M., Coffey M., Markert J.M. (2014). Phase 1 clinical trial of intratumoral reovirus infusion for the treatment of recurrent malignant gliomas in adults. Mol. Ther..

[75] Merrill M.K., Bernhardt G., Sampson J.H., Wikstrand C.J., Bigner D.D., Gromeier M. (2004). Poliovirus receptor CD155-targeted oncolysis of glioma. Neuro Oncol..

[76] Ochiai H., Campbell S.A., Archer G.E., Chewning T.A., Dragunsky E., Ivanov A., Gromeier M., Sampson J.H. (2006). Targeted therapy for glioblastoma multiforme neoplastic meningitis with intrathecal delivery of an oncolytic recombinant poliovirus. Clin. Cancer Res..

[77] Cello J., Toyoda H., Dejesus N., Dobrikova E.Y., Gromeier M., Wimmer E. (2008). Growth phenotypes and biosafety profiles in poliovirus-receptor transgenic mice of recombinant oncolytic polio/human rhinoviruses. J. Med. Virol..

[78] Dobrikova E.Y., Broadt T., Poiley-Nelson J., Yang X., Soman G., Giardina S., Harris R., Gromeier M. (2008). Recombinant oncolytic poliovirus eliminates glioma in vivo without genetic adaptation to a pathogenic phenotype. Mol. Ther..

[79] Jahan N., Wimmer E., Mueller S. (2011). A host-specific, temperature-sensitive translation defect determines the attenuation phenotype of a human rhinovirus/poliovirus chimera, PV1(RIPO). J. Virol..

[80] Holl E.K., Brown M.C., Boczkowski D., McNamara M.A., George D.J., Bigner D.D., Gromeier M., Nair S.K. (2016). Recombinant oncolytic poliovirus, PVSRIPO, has potent cytotoxic and innate inflammatory effects, mediating therapy in human breast and prostate cancer xenograft models. Oncotarget.

[81] Walton R.W., Brown M.C., Sacco M.T., Gromeier M. (2018). Engineered Oncolytic Poliovirus PVSRIPO Subverts MDA5-Dependent Innate Immune Responses in Cancer Cells. J. Virol..

[82] Msaouel P., Opyrchal M., Dispenzieri A., Peng K.W., Federspiel M.J., Russell S.J., Galanis E. (2018). Clinical Trials with Oncolytic Measles Virus: Current Status and Future Prospects. Curr. Cancer Drug Targets.

[83] Cloughesy T.F., Petrecca K., Walbert T., Butowski N., Salacz M., Perry J., Damek D., Bota D., Bettegowda C., Zhu J.J. (2020). Effect of Vocimagene Amiretrorepvec in Combination With Flucytosine vs Standard of Care on Survival Following Tumor Resection in Patients With Recurrent High-Grade Glioma: A Randomized Clinical Trial. JAMA Oncol..

[84] Yaacov B., Eliahoo E., Lazar I., Ben-Shlomo M., Greenbaum I., Panet A., Zakay-Rones Z. (2008). Selective oncolytic effect of an attenuated Newcastle disease virus (NDV-HUJ) in lung tumors. Cancer Gene Ther..

[85] Ferguson M.S., Lemoine N.R., Wang Y. (2012). Systemic delivery of oncolytic viruses: Hopes and hurdles. Adv. Virol..

[86] Ramachandran M., Yu D., Dyczynski M., Baskaran S., Zhang L., Lulla A., Lulla V., Saul S., Nelander S., Dimberg A. (2017). Safe and Effective Treatment of Experimental Neuroblastoma and Glioblastoma Using Systemically Delivered Triple MicroRNA-Detargeted Oncolytic Semliki Forest Virus. Clin. Cancer Res..

[87] Lun X.Q., Jang J.H., Tang N., Deng H., Head R., Bell J.C., Stojdl D.F., Nutt C.L., Senger D.L., Forsyth P.A. (2009). Efficacy of systemically administered oncolytic vaccinia virotherapy for malignant gliomas is enhanced by combination therapy with rapamycin or cyclophosphamide. Clin. Cancer Res..

[88] Advani S.J., Buckel L., Chen N.G., Scanderbeg D.J., Geissinger U., Zhang Q., Yu Y.A., Aguilar R.J., Mundt A.J., Szalay A.A. (2012). Preferential replication of systemically delivered oncolytic vaccinia virus in focally irradiated glioma xenografts. Clin. Cancer Res..

[89] Muik A., Stubbert L.J., Jahedi R.Z., Geibeta Y., Kimpel J., Dold C., Tober R., Volk A., Klein S., Dietrich U. (2014). Re-engineering vesicular stomatitis virus to abrogate neurotoxicity, circumvent humoral immunity, and enhance oncolytic potency. Cancer Res..

[90] Geletneky K., Kiprianova I., Ayache A., Koch R., Herrero Y.C.M., Deleu L., Sommer C., Thomas N., Rommelaere J., Schlehofer J.R. (2010). Regression of advanced rat and human gliomas by local or systemic treatment with oncolytic parvovirus H-1 in rat models. Neuro Oncol..

[91] Ruiz A.J., Hadac E.M., Nace R.A., Russell S.J. (2016). MicroRNA-Detargeted Mengovirus for Oncolytic Virotherapy. J. Virol..

[92] Liu Z., Zhao X., Mao H., Baxter P.A., Huang Y., Yu L., Wadhwa L., Su J.M., Adesina A., Perlaky L. (2013). Intravenous injection of oncolytic picornavirus SVV-001 prolongs animal survival in a panel of primary tumor-based orthotopic xenograft mouse models of pediatric glioma. Neuro Oncol..

[93] Zhang Q., Xiang W., Yi D.Y., Xue B.Z., Wen W.W., Abdelmaksoud A., Xiong N.X., Jiang X.B., Zhao H.Y., Fu P. (2018). Current status and potential challenges of mesenchymal stem cell-based therapy for malignant gliomas. Stem. Cell Res. Ther..

[94] Yong R.L., Shinojima N., Fueyo J., Gumin J., Vecil G.G., Marini F.C., Bogler O., Andreeff M., Lang F.F. (2009). Human bone marrow-derived mesenchymal stem cells for intravascular delivery of oncolytic adenovirus Delta24-RGD to human gliomas. Cancer Res..

[95] Ellis J.A., Banu M., Hossain S.S., Singh-Moon R., Lavine S.D., Bruce J.N., Joshi S. (2015). Reassessing the Role of Intra-Arterial Drug Delivery for Glioblastoma Multiforme Treatment. J. Drug Deliv..

[96] Srinivasan V.M., Lang F.F., Kan P. (2021). Intraarterial delivery of virotherapy for glioblastoma. Neurosurg. Focus.

[97] Shi M., Sanche L. (2019). Convection-Enhanced Delivery in Malignant Gliomas: A Review of Toxicity and Efficacy. J. Oncol..

[98] Sminia P., Westerman B.A. (2016). Blood-brain barrier crossing and breakthroughs in glioblastoma therapy. Br. J. Clin. Pharmacol..

[99] Majerova P., Michalicova A., Cente M., Hanes J., Vegh J., Kittel A., Kosikova N., Cigankova V., Mihaljevic S., Jadhav S. (2019). Trafficking of immune cells across the blood-brain barrier is modulated by neurofibrillary pathology in tauopathies. PLoS ONE.

[100] Marchetti L., Engelhardt B. (2020). Immune cell trafficking across the blood-brain barrier in the absence and presence of neuroinflammation. Vasc. Biol..

[101] Matias D., Balca-Silva J., da Graca G.C., Wanjiru C.M., Macharia L.W., Nascimento C.P., Roque N.R., Coelho-Aguiar J.M., Pereira C.M., Dos Santos M.F. (2018). Microglia/Astrocytes-Glioblastoma Crosstalk: Crucial Molecular Mechanisms and Microenvironmental Factors. Front. Cell Neurosci..

[102] Dunn G.P., Fecci P.E., Curry W.T. (2012). Cancer immunoediting in malignant glioma. Neurosurgery.

[103] Fornara O., Odeberg J., Wolmer Solberg N., Tammik C., Skarman P., Peredo I., Stragliotto G., Rahbar A., Soderberg-Naucler C. (2015). Poor survival in glioblastoma patients is associated with early signs of immunosenescence in the CD4 T-cell compartment after surgery. OncoImmunology.

[104] Woroniecka K., Chongsathidkiet P., Rhodin K., Kemeny H., Dechant C., Farber S.H., Elsamadicy A.A., Cui X., Koyama S., Jackson C. (2018). T-Cell Exhaustion Signatures Vary with Tumor Type and Are Severe in Glioblastoma. Clin. Cancer Res..

[105] Xu L., Xiao H., Xu M., Zhou C., Yi L., Liang H. (2011). Glioma-derived T cell immunoglobulin- and mucin domain-containing molecule-4 (TIM4) contributes to tumor tolerance. J. Biol. Chem..

[106] Fecci P.E., Mitchell D.A., Whitesides J.F., Xie W., Friedman A.H., Archer G.E., Herndon J.E., Bigner D.D., Dranoff G., Sampson J.H. (2006). Increased regulatory T-cell fraction amidst a diminished CD4 compartment explains cellular immune defects in patients with malignant glioma. Cancer Res..

[107] Vignali D.A., Collison L.W., Workman C.J. (2008). How regulatory T cells work. Nat. Rev. Immunol..

[108] Wing K., Sakaguchi S. (2010). Regulatory T cells exert checks and balances on self tolerance and autoimmunity. Nat. Immunol..

[109] Samuels V., Barrett J.M., Bockman S., Pantazis C.G., Allen M.B. (1989). Immunocytochemical study of transforming growth factor expression in benign and malignant gliomas. Am. J. Pathol..

[110] Wainwright D.A., Balyasnikova I.V., Chang A.L., Ahmed A.U., Moon K.S., Auffinger B., Tobias A.L., Han Y., Lesniak M.S. (2012). IDO expression in brain tumors increases the recruitment of regulatory T cells and negatively impacts survival. Clin. Cancer Res..

[111] Crane C.A., Han S.J., Barry J.J., Ahn B.J., Lanier L.L., Parsa A.T. (2010). TGF-beta downregulates the activating receptor NKG2D on NK cells and CD8+ T cells in glioma patients. Neuro Oncol..

[112] Lin A., Yan W.H. (2015). Human Leukocyte Antigen-G (HLA-G) Expression in Cancers: Roles in Immune Evasion, Metastasis and Target for Therapy. Mol. Med..

[113] Ralainirina N., Poli A., Michel T., Poos L., Andres E., Hentges F., Zimmer J. (2007). Control of NK cell functions by CD4+CD25+ regulatory T cells. J. Leukoc. Biol..

[114] Alban T.J., Alvarado A.G., Sorensen M.D., Bayik D., Volovetz J., Serbinowski E., Mulkearns-Hubert E.E., Sinyuk M., Hale J.S., Onzi G.R. (2018). Global immune fingerprinting in glioblastoma patient peripheral blood reveals immune-suppression signatures associated with prognosis. JCI Insight.

[115] Chai E., Zhang L., Li C. (2019). LOX-1+ PMN-MDSC enhances immune suppression which promotes glioblastoma multiforme progression. Cancer Manag. Res..

[116] Nishie A., Ono M., Shono T., Fukushi J., Otsubo M., Onoue H., Ito Y., Inamura T., Ikezaki K., Fukui M. (1999). Macrophage infiltration and heme oxygenase-1 expression correlate with angiogenesis in human gliomas. Clin. Cancer Res..

[117] Mieczkowski J., Kocyk M., Nauman P., Gabrusiewicz K., Sielska M., Przanowski P., Maleszewska M., Rajan W.D., Pszczolkowska D., Tykocki T. (2015). Down-regulation of IKKbeta expression in glioma-infiltrating microglia/macrophages is associated with defective inflammatory/immune gene responses in glioblastoma. Oncotarget.

[118] Russell L., Peng K.W., Russell S.J., Diaz R.M. (2019). Oncolytic Viruses: Priming Time for Cancer Immunotherapy. BioDrugs.

[119] Russell S.J., Peng K.W. (2017). Oncolytic Virotherapy: A Contest between Apples and Oranges. Mol. Ther..

[120] Zhang Q., Liu F. (2020). Advances and potential pitfalls of oncolytic viruses expressing immunomodulatory transgene therapy for malignant gliomas. Cell Death Dis..

[121] Liu P., Wang Y., Wang Y., Kong Z., Chen W., Li J., Chen W., Tong Y., Ma W., Wang Y. (2020). Effects of oncolytic viruses and viral vectors on immunity in glioblastoma. Gene Ther..

[122] Russell S.J., Barber G.N. (2018). Oncolytic Viruses as Antigen-Agnostic Cancer Vaccines. Cancer Cell.

[123] Zhang T., Kordish D.H., Suryawanshi Y.R., Eversole R.R., Kohler S., Mackenzie C.D., Essani K. (2018). Oncolytic Tanapoxvirus Expressing Interleukin-2 is Capable of Inducing the Regression of Human Melanoma Tumors in the Absence of T Cells. Curr. Cancer Drug Targets.

[124] Ekeke C.N., Russell K.L., Murthy P., Guo Z.S., Soloff A.C., Weber D., Pan W., Lotze M.T., Dhupar R. (2020). Intrapleural interleukin-2-expressing oncolytic virotherapy enhances acute antitumor effects and T-cell receptor diversity in malignant pleural disease. J. Thorac. Cardiovasc. Surg..

[125] Heinio C., Havunen R., Santos J., de Lint K., Cervera-Carrascon V., Kanerva A., Hemminki A. (2020). TNFa and IL2 Encoding Oncolytic Adenovirus Activates Pathogen and Danger-Associated Immunological Signaling. Cells.

[126] Saha D., Martuza R.L., Rabkin S.D. (2017). Macrophage Polarization Contributes to Glioblastoma Eradication by Combination Immunovirotherapy and Immune Checkpoint Blockade. Cancer Cell.

[127] Parker J.N., Gillespie G.Y., Love C.E., Randall S., Whitley R.J., Markert J.M. (2000). Engineered herpes simplex virus expressing IL-12 in the treatment of experimental murine brain tumors. Proc. Natl. Acad. Sci. USA.

[128] Tang B., Guo Z.S., Bartlett D.L., Yan D.Z., Schane C.P., Thomas D.L., Liu J., McFadden G., Shisler J.L., Roy E.J. (2020). Synergistic Combination of Oncolytic Virotherapy and Immunotherapy for Glioma. Clin. Cancer Res..

[129] Andreansky S., He B., van Cott J., McGhee J., Markert J.M., Gillespie G.Y., Roizman B., Whitley R.J. (1998). Treatment of intracranial gliomas in immunocompetent mice using herpes simplex viruses that express murine interleukins. Gene Ther..

[130] Lun X., Chan J., Zhou H., Sun B., Kelly J.J., Stechishin O.O., Bell J.C., Parato K., Hu K., Vaillant D. (2010). Efficacy and safety/toxicity study of recombinant vaccinia virus JX-594 in two immunocompetent animal models of glioma. Mol. Ther..

[131] Herrlinger U., Jacobs A., Quinones A., Woiciechowsky C., Sena-Esteves M., Rainov N.G., Fraefel C., Breakefield X.O. (2000). Helper virus-free herpes simplex virus type 1 amplicon vectors for granulocyte-macrophage colony-stimulating factor-enhanced vaccination therapy for experimental glioma. Hum. Gene Ther..

[132] Suryawanashi Y.R., Zhang T., Woyczesczyk H.M., Christie J., Byers E., Kohler S., Eversole R., Mackenzie C., Essani K. (2017). T-independent response mediated by oncolytic tanapoxvirus recombinants expressing interleukin-2 and monocyte chemoattractant protein-1 suppresses human triple negative breast tumors. Med. Oncol..

[133] Eckert E.C., Nace R.A., Tonne J.M., Evgin L., Vile R.G., Russell S.J. (2020). Generation of a Tumor-Specific Chemokine Gradient Using Oncolytic Vesicular Stomatitis Virus Encoding CXCL9. Mol. Ther. Oncolytics.

[134] Kobayashi M., Fitz L., Ryan M., Hewick R.M., Clark S.C., Chan S., Loudon R., Sherman F., Perussia B., Trinchieri G. (1989). Identification and purification of natural killer cell stimulatory factor (NKSF), a cytokine with multiple biologic effects on human lymphocytes. J. Exp. Med..

[135] Hsieh C.S., Macatonia S.E., Tripp C.S., Wolf S.F., O’Garra A., Murphy K.M. (1993). Development of TH1 CD4+ T cells through IL-12 produced by Listeria-induced macrophages. Science.

[136] Manetti R., Parronchi P., Giudizi M.G., Piccinni M.P., Maggi E., Trinchieri G., Romagnani S. (1993). Natural killer cell stimulatory factor (interleukin 12 [IL-12]) induces T helper type 1 (Th1)-specific immune responses and inhibits the development of IL-4-producing Th cells. J. Exp. Med..

[137] Trinchieri G. (1998). Interleukin-12: A cytokine at the interface of inflammation and immunity. Adv. Immunol..

[138] Croft M. (2010). Control of immunity by the TNFR-related molecule OX40 (CD134). Annu. Rev. Immunol..

[139] Frentsch M., Stark R., Matzmohr N., Meier S., Durlanik S., Schulz A.R., Stervbo U., Jurchott K., Gebhardt F., Heine G. (2013). CD40L expression permits CD8+ T cells to execute immunologic helper functions. Blood.

[140] Chaurasiya S., Chen N.G., Warner S.G. (2018). Oncolytic Virotherapy versus Cancer Stem Cells: A Review of Approaches and Mechanisms. Cancers.

[141] Jiang H., Gomez-Manzano C., Aoki H., Alonso M.M., Kondo S., McCormick F., Xu J., Kondo Y., Bekele B.N., Colman H. (2007). Examination of the therapeutic potential of Delta-24-RGD in brain tumor stem cells: Role of autophagic cell death. J. Natl. Cancer Inst..

[142] Fulci G., Dmitrieva N., Gianni D., Fontana E.J., Pan X., Lu Y., Kaufman C.S., Kaur B., Lawler S.E., Lee R.J. (2007). Depletion of peripheral macrophages and brain microglia increases brain tumor titers of oncolytic viruses. Cancer Res..

[143] Denton N.L., Chen C.Y., Scott T.R., Cripe T.P. (2016). Tumor-Associated Macrophages in Oncolytic Virotherapy: Friend or Foe?. BioMedicines.

[144] Schneider-Schaulies J. (2000). Cellular receptors for viruses: Links to tropism and pathogenesis. J. Gen. Virol..

[145] Nomaguchi M., Fujita M., Miyazaki Y., Adachi A. (2012). Viral tropism. Front. Microbiol..

[146] Zhang W., Fulci G., Wakimoto H., Cheema T.A., Buhrman J.S., Jeyaretna D.S., Stemmer Rachamimov A.O., Rabkin S.D., Martuza R.L. (2013). Combination of oncolytic herpes simplex viruses armed with angiostatin and IL-12 enhances antitumor efficacy in human glioblastoma models. Neoplasia.

[147] Castro M.G., Candolfi M., Wilson T.J., Calinescu A., Paran C., Kamran N., Koschmann C., Moreno-Ayala M.A., Assi H., Lowenstein P.R. (2014). Adenoviral vector-mediated gene therapy for gliomas: Coming of age. Expert Opin. Biol. Ther..

[148] Manikandan C., Kaushik A., Sen D. (2020). Viral vector: Potential therapeutic for glioblastoma multiforme. Cancer Gene Ther..

[149] Paun A., Pitha P.M. (2007). The innate antiviral response: New insights into a continuing story. Adv. Virus Res..

[150] Lemos de Matos A., Franco L.S., McFadden G. (2020). Oncolytic Viruses and the Immune System: The Dynamic Duo. Mol. Ther. Methods Clin. Dev..

[151] Kurokawa C., Iankov I.D., Anderson S.K., Aderca I., Leontovich A.A., Maurer M.J., Oberg A.L., Schroeder M.A., Giannini C., Greiner S.M. (2018). Constitutive Interferon Pathway Activation in Tumors as an Efficacy Determinant Following Oncolytic Virotherapy. J. Natl. Cancer Inst..

[152] Meisen W.H., Wohleb E.S., Jaime-Ramirez A.C., Bolyard C., Yoo J.Y., Russell L., Hardcastle J., Dubin S., Muili K., Yu J. (2015). The Impact of Macrophage- and Microglia-Secreted TNFalpha on Oncolytic HSV-1 Therapy in the Glioblastoma Tumor Microenvironment. Clin. Cancer Res..

[153] Butte M.J., Pena-Cruz V., Kim M.J., Freeman G.J., Sharpe A.H. (2008). Interaction of human PD-L1 and B7-1. Mol. Immunol..

[154] Krummel M.F., Allison J.P. (1995). CD28 and CTLA-4 have opposing effects on the response of T cells to stimulation. J. Exp. Med..

[155] Nduom E.K., Wei J., Yaghi N.K., Huang N., Kong L.Y., Gabrusiewicz K., Ling X., Zhou S., Ivan C., Chen J.Q. (2016). PD-L1 expression and prognostic impact in glioblastoma. Neuro Oncol..

[156] Romani M., Pistillo M.P., Carosio R., Morabito A., Banelli B. (2018). Immune Checkpoints and Innovative Therapies in Glioblastoma. Front. Oncol..

[157] Brahm C.G., van Linde M.E., Enting R.H., Schuur M., Otten R.H.J., Heymans M.W., Verheul H.M.W., Walenkamp A.M.E. (2020). The Current Status of Immune Checkpoint Inhibitors in Neuro-Oncology: A Systematic Review. Cancers.

[158] Galstyan A., Markman J.L., Shatalova E.S., Chiechi A., Korman A.J., Patil R., Klymyshyn D., Tourtellotte W.G., Israel L.L., Braubach O. (2019). Blood-brain barrier permeable nano immunoconjugates induce local immune responses for glioma therapy. Nat. Commun..

[159] Erel-Akbaba G., Carvalho L.A., Tian T., Zinter M., Akbaba H., Obeid P.J., Chiocca E.A., Weissleder R., Kantarci A.G., Tannous B.A. (2019). Radiation-Induced Targeted Nanoparticle-Based Gene Delivery for Brain Tumor Therapy. ACS Nano.

[160] Lauko A., Lo A., Ahluwalia M.S., Lathia J.D. (2021). Cancer cell heterogeneity & plasticity in glioblastoma and brain tumors. Semin. Cancer Biol..

[161] Akgul S., Patch A.M., D’Souza R.C.J., Mukhopadhyay P., Nones K., Kempe S., Kazakoff S.H., Jeffree R.L., Stringer B.W., Pearson J.V. (2019). Intratumoural Heterogeneity Underlies Distinct Therapy Responses and Treatment Resistance in Glioblastoma. Cancers.

[162] Qazi M.A., Vora P., Venugopal C., Sidhu S.S., Moffat J., Swanton C., Singh S.K. (2017). Intratumoral heterogeneity: Pathways to treatment resistance and relapse in human glioblastoma. Ann. Oncol..

[163] van den Hengel S.K., Balvers R.K., Dautzenberg I.J., van den Wollenberg D.J., Kloezeman J.J., Lamfers M.L., Sillivis-Smit P.A., Hoeben R.C. (2013). Heterogeneous reovirus susceptibility in human glioblastoma stem-like cell cultures. Cancer Gene Ther..

[164] Lamfers M.L., Idema S., Bosscher L., Heukelom S., Moeniralm S., van der Meulen-Muileman I.H., Overmeer R.M., van der Valk P., van Beusechem V.W., Gerritsen W.R. (2007). Differential effects of combined Ad5- delta 24RGD and radiation therapy in in vitro versus in vivo models of malignant glioma. Clin. Cancer Res..

[165] Grill J., Van Beusechem V.W., Van Der Valk P., Dirven C.M., Leonhart A., Pherai D.S., Haisma H.J., Pinedo H.M., Curiel D.T., Gerritsen W.R. (2001). Combined targeting of adenoviruses to integrins and epidermal growth factor receptors increases gene transfer into primary glioma cells and spheroids. Clin. Cancer Res..

[166] Tamura K., Wakimoto H., Agarwal A.S., Rabkin S.D., Bhere D., Martuza R.L., Kuroda T., Kasmieh R., Shah K. (2013). Multimechanistic tumor targeted oncolytic virus overcomes resistance in brain tumors. Mol. Ther..

[167] Ulasov I.V., Rivera A.A., Sonabend A.M., Rivera L.B., Wang M., Zhu Z.B., Lesniak M.S. (2007). Comparative evaluation of survivin, midkine and CXCR4 promoters for transcriptional targeting of glioma gene therapy. Cancer Biol. Ther..

[168] Post D.E., Sandberg E.M., Kyle M.M., Devi N.S., Brat D.J., Xu Z., Tighiouart M., Van Meir E.G. (2007). Targeted cancer gene therapy using a hypoxia inducible factor dependent oncolytic adenovirus armed with interleukin-4. Cancer Res..

[169] Kambara H., Okano H., Chiocca E.A., Saeki Y. (2005). An oncolytic HSV-1 mutant expressing ICP34.5 under control of a nestin promoter increases survival of animals even when symptomatic from a brain tumor. Cancer Res..

[170] Spencer D.A., Young J.S., Kanojia D., Kim J.W., Polster S.P., Murphy J.P., Lesniak M.S. (2015). Unlocking the promise of oncolytic virotherapy in glioma: Combination with chemotherapy to enhance efficacy. Ther. Deliv..

[171] O’Cathail S.M., Pokrovska T.D., Maughan T.S., Fisher K.D., Seymour L.W., Hawkins M.A. (2017). Combining Oncolytic Adenovirus with Radiation-A Paradigm for the Future of Radiosensitization. Front. Oncol..

[172] Conry R.M., Westbrook B., McKee S., Norwood T.G. (2018). Talimogene laherparepvec: First in class oncolytic virotherapy. Hum. Vaccin Immunother..

[173] Martikainen M., Essand M. (2019). Virus-Based Immunotherapy of Glioblastoma. Cancers.

